# The natural history of neurolymphomatosis

**DOI:** 10.1038/s44276-024-00053-x

**Published:** 2024-04-23

**Authors:** Elizabeth Xu, Quan Ho, Ashley Liu, Shiva Gautam, Eric T. Wong

**Affiliations:** 1 Department of Chemistry, University of Pennsylvania, Philadelphia, PA USA; 2 Department of Biomedical Engineering, Boston University, Boston, MA USA; 3 Beth Israel Deaconess Medical Center & Harvard Medical School, Boston, MA USA; 4 Department of Biology, Boston University, Boston, MA USA; 5 Department of Biostatistics, University of Florida at Gainesville, Gainesville, FL USA; 6 Division of Hematology/Oncology, Rhode Island Hospital & The Warren Alpert Medical School of Brown University, Providence, RI USA

## Abstract

**Background:**

Neurolymphomatosis is a lymphoid malignancy of the peripheral nervous system and its natural history is poorly understood.

**Methods:**

We performed PubMed search and extracted clinical data for Kaplan-Meier statistics to determine outcome parameters over time. Kruskal-Wallis test was performed to compare prognostic factors.

**Results:**

Our search identified 559 patients and their median age was 61 years. Median overall survival (OS) was 12.0 (range 10.0–15.0) months. Diffuse large B-cell lymphoma was the most frequent histology, involving the brachial plexus, cranial nerves, and sciatic nerve. None had molecular profiling. There was a progressive lengthening of OS in successive decades, from 0.5 (95% CI 0.0–0.8) to 26.4 (95% CI 18.0–34.8) months between 1951 and 2022 (r^2^ = 0.0528, *p* < 0.00001). Time from first treatment (treatment 1) to progression increased from 2.0 to 36.0 (95% CI 6.5–50.7) months (r^2^ = 0.0961, *p* = 0.00236). Time from symptom onset to diagnosis remained unchanged (r^2^ = 0.0000556, *p* = 0.939). Patients were most frequently treated with methotrexate, rituximab, and/or radiation either alone or in combination. Primary neurolymphomatosis had a better prognosis than secondary neurolymphomatosis. No OS difference was noted between B- and T-cell disease, but low-grade B-cell performed better than Burkitt’s lymphoma.

**Discussion:**

Better outcome for patients with neurolymphomatosis is noted over time. But timely diagnosis remains a major problem that needs improvement.

## Introduction

Neurolymphomatosis (NL) is a subtype of extranodal lymphoma that invades the peripheral nervous system [1]. It was first described by Marek et al. in chickens as fowl paralysis caused by tumor-like, lymphoid-cell infiltration of multiple plexi and peripheral nerves, and later found to be associated with a cytomegalovirus-like herpes virus [2, 3]. In humans, the predominant histology is CD20 + B-cell non-Hodgkin’s lymphoma with a high proliferation index [1]. NL associated with T cells is rare. The presenting symptoms include motor and/or sensory dysfunctions with or without pain [1]. This malignancy is considered primary when it is found exclusively in a peripheral nerve, and it is denoted as secondary when the lymphoma spreads from other organs to the peripheral nervous system.

The natural history of NL is unknown. The rarity of this malignancy precludes a large-scale epidemiological analysis. Only two large series were published. The International Primary CNS Lymphoma Collaborative Group reported in 2010 a retrospective analysis of 50 patients with NL and found a median overall survival (OS) of 10 months [4]. Peripheral nerve, cranial nerve, plexus, or a combination was the predominant site of involvement, and this cohort was treated with high-dose methotrexate (HD-MTX), intra-cerebrospinal fluid chemotherapy and radiotherapy [4]. In 2021, a single-institution report of 40 patients compared treatment with or without rituximab, and the authors concluded that those treated with rituximab had significantly longer OS and progression-free survival (PFS) [5]. Still, the literature of NL is populated by case reports or small series of patients diagnosed at post-mortem, and treated patients in these two reports may not be representative of the general population. Therefore, we undertook this meta-analysis of published literature to define these patients’ presenting symptoms, survival, prognostic factors, and treatment outcome.

## Methods

**Fig. 1:**
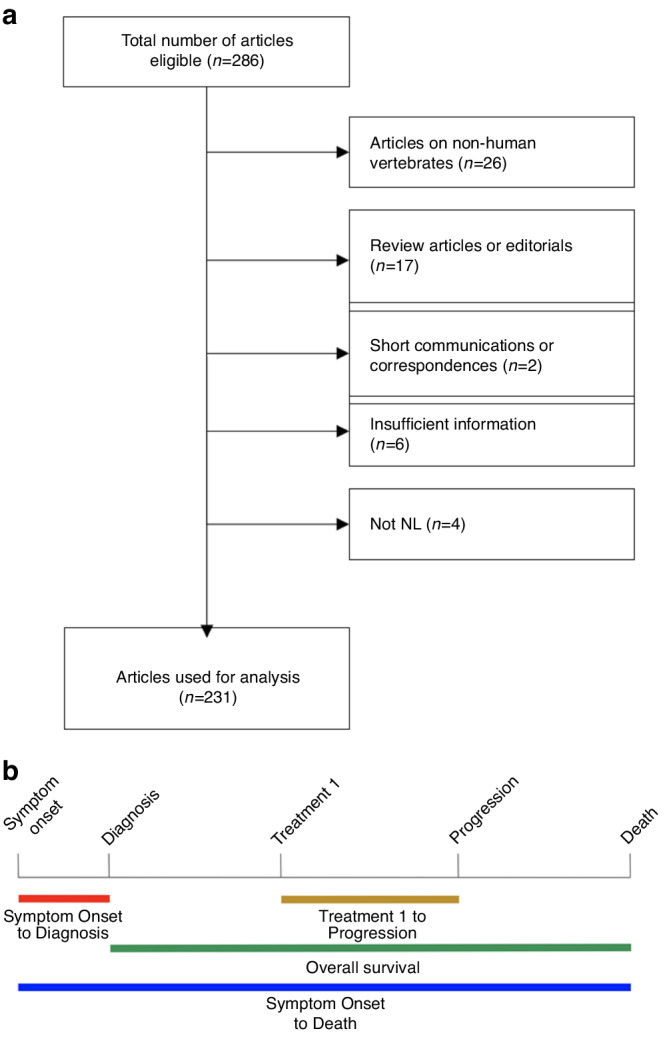
Consort diagram and outcome parameters. **a** Consort diagram for article inclusion and exclusion. **b** Graphical depiction of outcome parameters: (i) OS (green), (ii) time from treatment 1 to progression (gold), (iii) time from symptom onset to diagnosis (red), and (iv) time from symptom onset to death (blue).

PubMed searches of the term “neurolymphomatosis” were performed, and the content of each article was review by EX and ETW (Fig. 1a). Most of the articles were written in English, but for those published in Japanese (15), French (7), Spanish (5), German (2), and Chinese (2), data were extracted by recruited native speakers of these languages. The following data were collected from the publications: (i) year of publication, (ii) age, (iii) gender, (iv) type of lymphoma, (v) primary or secondary NL, (vi) nerves infiltrated by lymphoma, (vii) modality of diagnosis such as magnetic resonance imaging (MRI), computed tomography (CT), [^18^F]fluorodeoxyglucose-positron emission tomography (FDG-PET), or tissue biopsy, (viii) treatment received, and (ix) survival times. Missing data were not imputed in the final analysis due to heterogeneity of published information.

Statistical analyses were performed in R version 4.2.3 using packages: ggplot2, plyr, survival, survminer, prodlim, Hmisc, pracma, readxl and ggpubr. A fixed-effect model was applied and Kaplan–Meier statistics were used to determine outcome parameters over time. Four major outcome parameters were determined: (i) time from diagnosis to death or overall survival (OS), (ii) time from treatment 1 (first treatment) to progression, (iii) time from symptom onset to diagnosis, and (iv) time from symptom onset to death (periods i + iii) (Fig. 1b). Prognostic factors were identified according to (i) age, (ii) types of lymphoma, and (iii) locations of NL. Kruskal-Wallis test was performed to determine differences among groups, with Bonferroni adjustment to minimize false positives from multiple testing. Statistical significance was defined at *p* ≤ 0.05.

## Results

### Patient demographics

We were able to identify 286 articles and excluded those describing NL in non-human vertebrates (*n* = 26), reviews (*n* = 17), short communications or correspondences with incomplete clinical data (*n* = 2), and publications that had insufficient clinical information (*n* = 6) or were deemed unrelated to NL (*n* = 4) (Fig. 1a). From the remaining 231 articles, 559 cases of NL were extracted from articles published between 1951 and 2022 (Supplementary Table 1). There were 326 males and 222 females, while 11 had unspecified gender information. The median age of the cohort was 61 (range 2–92 years). There were 541 adults at age ≥18 years, 3 adolescents from age 13–17, 1 child at age 8, 1 toddler at age 2, and 13 with unspecified age.

### Diagnosis and site of involvement

One hundred fifty-four patients developed primary NL and 329 acquired secondary NL after a diagnosis of systemic lymphoma, while 76 were unclassifiable due to insufficient information. Diagnosis was established in 267 patients by tissue biopsy of a nerve, 258 by radiology only, 1 by lumbar puncture, 2 by flow cytometry of vitreous fluid, 40 by autopsy, and 2 unknowns. Only 7% had electromyography and/or nerve conduction study. The sites of involvement are listed in Supplementary Table 2. The most common site is brachial plexus (*n* = 142), followed by unspecified cranial nerves (*n* = 128), and the sciatic nerve (*n* = 125).

From a histology perspective, diffuse large cell lymphoma (*n* = 347) was most common, followed by unspecified T-cell lymphoma (*n* = 19), and unspecified B cell lymphoma (*n* = 18) (Supplementary Table 3). None had molecular profiling. Fourteen patients from 12 articles had leukemias (2 acute non-lymphocytic leukemia, 2 adult T-cell leukemia/lymphoma, 7 chronic lymphocytic leukemia/small lymphocytic lymphoma, 1 pre-B acute lymphoblastic leukemia, 1 T-cell acute lymphocytic leukemia, and 1 T-cell lymphocytic leukemia). We included them in our study because leukemia and lymphomas are closely related due to their common underlying genetic variables that make certain leukemias behave like or transform into lymphomas.

### Survival characteristics and treatment outcomes

**Fig. 2:**
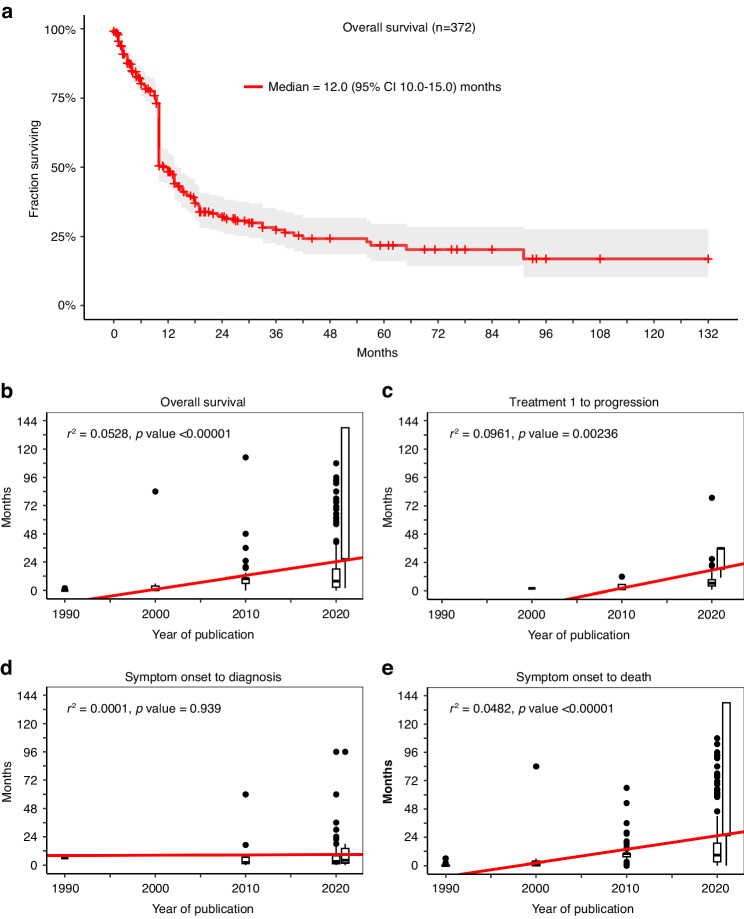
Survival in the entire NL population and stratified by decade of publication. **a** Median OS for the entire NL population was 12.0 (95% CI 10.0–15.0) months. Decade analyses were done from <1990–2022. **b** Median OS increased from a median of 0 (95% CI 0–1.6) to 26.2 (95% CI 0–189.4) months. **c** Median time from treatment 1 to progression also increased from 2 to 36.0 (95% CI 6.5–50.7) months. **d** Median time from symptoms onset to diagnosis stayed flat, 6.0 to 4.0 (95% CI 0–88.0) months. **e** Median time from symptoms onset to death increased from 0 (95% CI 0–5.3) to 26.2 (95% CI 5.3–188.0) months. See also Supplementary Table 4A–D.

We excluded 206 patients with undisclosed survival times and duration of treatment intervals. The OS for the cohort (*n* = 372) was 12.0 months (95% CI 10.0–15.0) (Fig. 2a). There was no difference between male and female, and their respective OS was 12.0 (95% CI 10.0–15.0) and 10.0 months (95% CI 10.0–18.9) (Kaplan-Meier not shown, *p* = 0.34768).

We then analyzed whether advances in diagnosis and treatment would prolong patient survival by grouping the publication dates into successive decades from 1950 to 2022. The decades from 1950 to 1990 were grouped together due to few reports (*n* = 7), while 2021–2022 were grouped together because of a higher number of patients in the publications (*n* = 8). As expected, there was a progressive lengthening of OS in each successive decades, from a median of 0 (95% CI 0–1.6) to 26.2 (95% CI 0–189.4) months from 1951 to 2022 (r^2^ = 0.0528, *p* < 0.00001) (Fig. 2b and Supplementary Table 4). This is probably attributed to treatment improvement since there was a progressive lengthening of time from treatment 1 to progression, from 2.0 (95% CI N/A) to 36.0 (95% CI 6.5–50.7) months (r^2^ = 0.0961, *p* = 0.00236) (Fig. 2c). However, timely diagnosis of NL remains difficult and time from symptom onset to diagnosis remains unchanged over decades (r^2^ = 0.0000556, *p* = 0.939) (Fig. 2d), but this does not influence time from symptom onset to death, which is combined from (i) time from symptom onset to diagnosis and (ii) OS (r^2^ = 0.00482, *p* < 0.00001) (Fig. 2e). Furthermore, about 7.2% of the NL population (*n* = 40) had a diagnosis established at autopsy and this trend did not decrease, but actually increased, over time (r^2^ = 0.555556), supporting the notion that this disease is extremely difficult to diagnose pre-mortem (Supplementary Fig. 1).

**Fig. 3:**
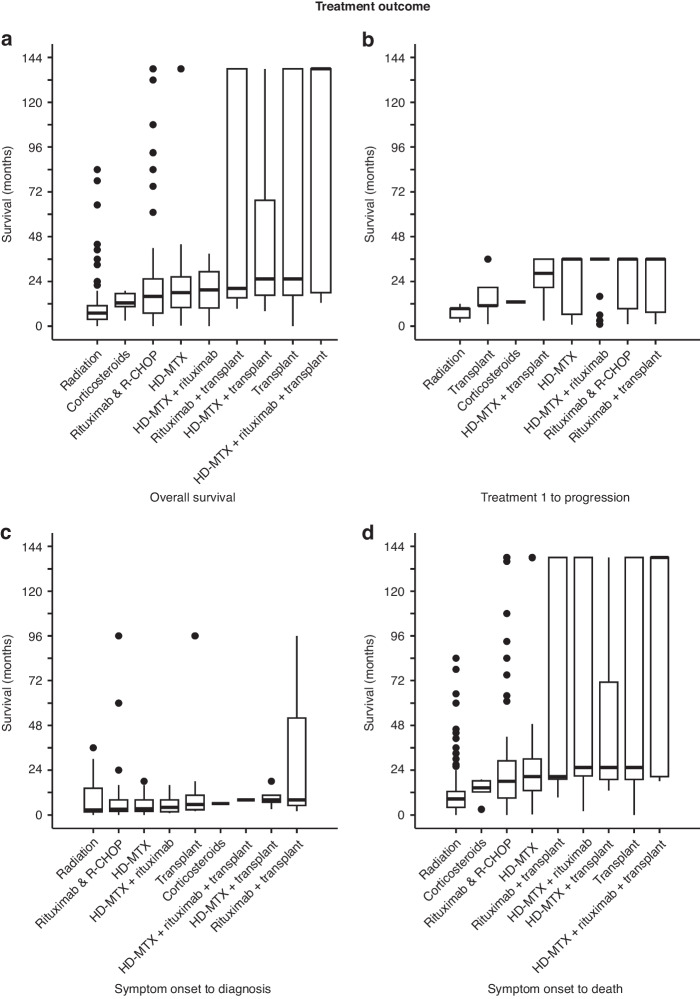
Kruskal-Wallis analysis of survival according to treatment regimens. **a** Analysis of OS showed a chi-squared value of 63.493 with 8 degrees of freedom and *p* < 0.00001. **b** Analysis of time from treatment 1 to progression showed a chi-squared value of 26.8 with 7 degrees of freedom and *p* = 0.00036. **c** Analysis of time from symptom onset to diagnosis showed a chi-squared value of 3.9978 with 8 degrees of freedom and *p* = 0.8573. **d** Analysis of time from symptom onset to death showed a chi-squared value of 69.849 with 8 degrees of freedom and *p* < 0.00001. See also Supplementary Table 5A–D.

We next performed a deeper analysis on the impact of treatment on patient survival. The most common regimen used was high-dose methotrexate (HD-MTX) alone or methotrexate-based combination regimens (*n* = 81), followed by rituximab alone or in combination with chemotherapy agent(s) (*n* = 176), and then radiation (*n* = 79). The treatment categories were divided into 9 subgroups for Kruskal-Wallis analysis, including (i) corticosteroid alone, (ii) HD-MTX alone, (iii) rituximab-based therapies, (iv) radiation alone, (v) HD-MTX + rituximab-based treatment, (vi) transplant, (vii) HD-MTX + transplant, (viii) rituximab-based therapy + transplant, and (ix) HD-MTX + rituximab-based therapy + transplant. There was high variance in OS among these 9 categories (*p* < 0.0001, Fig. 3a). Greatest difference was seen between radiation and transplant-based drug regimens, with the latter resulted in a longer OS compared to radiation (Supplementary Table 5A). High variance was also noted in time from treatment 1 to progression (*p* = 0.00036, Fig. 3b), and patients who received radiation alone had a shorter progression-free survival compared to those treated with medical therapies (Supplementary Table 5B). This suggests that NL should be viewed as a systemic malignancy rather than localized disease.

We next explored the relationship between time from symptom onset to diagnosis, which is a noteworthy period due to the extreme difficulty in diagnosing NL, and asked whether this has any relationship to the types of treatment patients received. Kruskal-Wallis analysis did not yield any significant difference among treatment groups (*p* = 0.8573, Fig. 3c), and *t*-test analyses between paired treatment categories were non-significant (Supplementary Table 5C), suggesting that timing of diagnosis did not influence subsequent treatments. We then analyzed the period from symptom onset to death and noted significant variance (*p* < 0.00001, Fig. 3d). Similar to the Kruskal-Wallis analysis for OS, patients treated with radiation had a significantly shortened survival (Supplementary Table 5D).

Sixty-two patients received a second treatment regimen but only 5 had progression-free survival data, and their disease was controlled for 3+, 5+, 6+, 15, and 19 months [6–9]. The regimens used include (i) HD-MTX [6], (ii) rituximab, ifosfamide, carboplatin, and etoposide (R-ICE) [7], (iii) HD-MTX and bone marrow transplant after confirmed negative PET study [8], (iv) salvage radiotherapy [9], and (v) cisplatin, cytarabine and dexamethasone [9].

Thirteen patients had survival data after receiving 3 (*n* = 11) or more (*n* = 2) regimens. An outlier who survived for 11 months received 4 regimens consisting of (i) cyclophosphamide, doxorubicin, vincristine and prednisone (CHOP), (ii) intrathecal cytarabine, intrathecal methotrexate, cytarabine, methotrexate and idarubicin (R-IDARAM), (iii) ifosfamide, etoposide, and radiation therapy, and (iv) repeat local radiotherapy [10]. Another outlier lived 42 months and received 6 regimens, consisting of (i) R-CHOP plus intrathecal MTX, (ii) MTX, cytarabine, and cyclophosphamide with total body irradiation as conditioning regimen for autologous stem cell transplant, (iii) rituximab, dexamethasone, cytarabine, and cisplatin (R-DHAP) followed by an allogeneic bone marrow transplant, (iv) local radiotherapy and donor lymphocyte infusion, followed by (v) rituximab, gemcitabine and oxaliplatin (R-GEM OX) in the fifth after a widespread relapse, and (vi) single-agent lenalidomide in the sixth regimen for salvage after progression [10].

### Prognostic factors

**Fig. 4:**
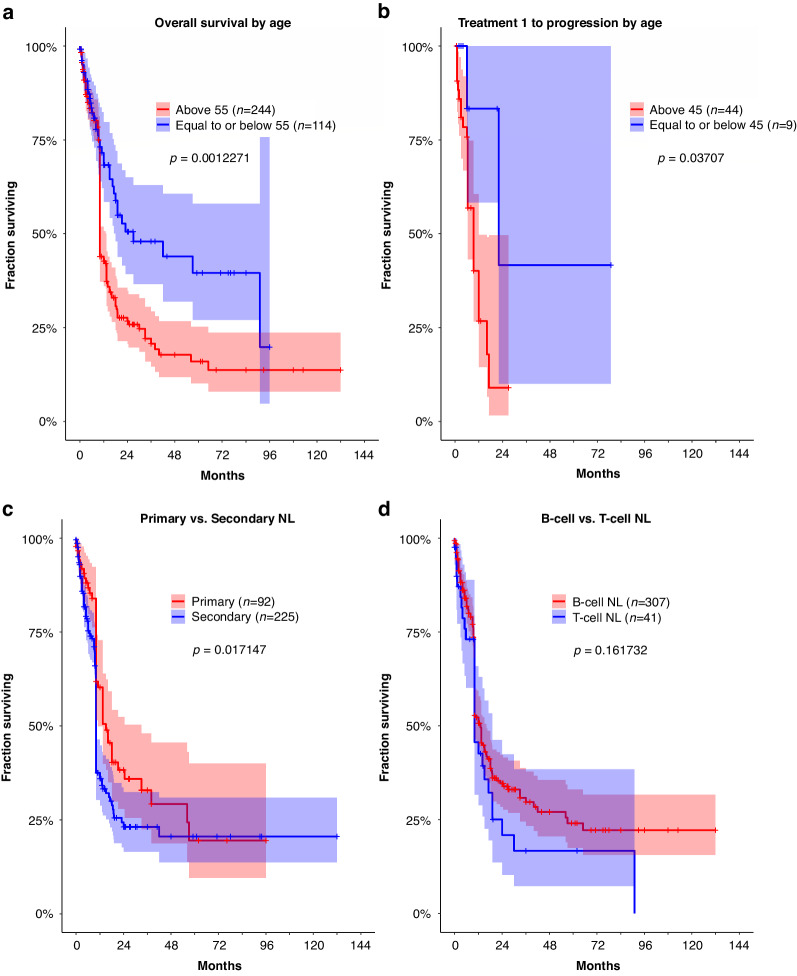
Survival of NL patients according to prognostic factors. **a** OS dichotomized at age 55, with a median OS of 23.0 (95% CI 17.1–N/A) months for patients at age ≤55 years and 10.0 (95% CI 10.0–13.4) months for those at age >55 years (*p* = 0.01227). **b** Time from treatment 1 to progression dichotomized at age 45, with a median of 12.0 (95% CI 6.0–22.0) months for patients at age ≤45 years versus 6.4 months (95% CI 6.4–9.4) months for those at age >45 years (*p* = 0.03707). **c** Median OS was 15.0 (95% CI 11.0–33.0) months for patients with primary NL and 10.0 (95% CI 10.0–10.0) months for those with secondary NL (*p* = 0.01715). **d** No difference in OS between B- and T-cell lymphomas, median OS 13.0 (95% CI 10.0–16.0) compared to 10.0 (95% CI 10.0–19.0) months, respectively (*p* = 0.16173).

We examined different potential prognostic factors that may influence patient survival. Because age is a strong prognostic factor for non-Hodgkin’s lymphoma [11], we first determined the optimal cutoff that would yield the greatest difference in survival. A significant difference in OS was noted among patients at age ≤55 (*n* = 114) versus those >55 (*n* = 244) years with a median of 27.0 (95% CI 18.0–N/A) and 10.0 (95% CI 10.0–13.4) months, respectively (*p* = 0.01227, Fig. 4a). In the cohort treated for NL, patients at age ≤45 years (*n* = 9) had a longer time from treatment 1 to progression compared to those >45 years (*n* = 44), median 22.0 (95% CI 22.0–N/A) versus 6.4 months (95% CI 6.4–12.0) months, respectively (*p* = 0.03707, Fig. 4b).

We next investigated whether the presentation or subtype of lymphoma would impact survival. First, patients with primary NL (*n* = 92) lived longer than those with secondary NL (*n* = 225), median OS 15.0 (95% CI 11.0–33.0) versus 10.0 (95% CI 10.0–10.0) months, respectively (*p* = 0.01715, Fig. 4c). Second, we compared patients with B- (*n* = 307) versus T-cell (*n* = 41) lymphomas (Supplementary Table 2) and found no difference in their OS, median 13.0 (95% CI 10.0–16.0) versus 10.0 (95% CI 10.0–19.0) months, respectively (*p* = 0.16173, Fig. 4d). The most frequently diagnosed histologies were diffuse large B-cell lymphoma (*n* = 360), unspecified T-cell lymphoma (*n* = 19), unspecified B-cell lymphoma (*n* = 18), and lymphoplasmatic lymphoma (Waldenstrom’s macroglobulinemia) (*n* = 14). Few patients (*n* = 18) had unspecified lymphoma subtype, and therefore they were excluded from subsequent analyses on prognosis according to specific histologies.

**Fig. 5:**
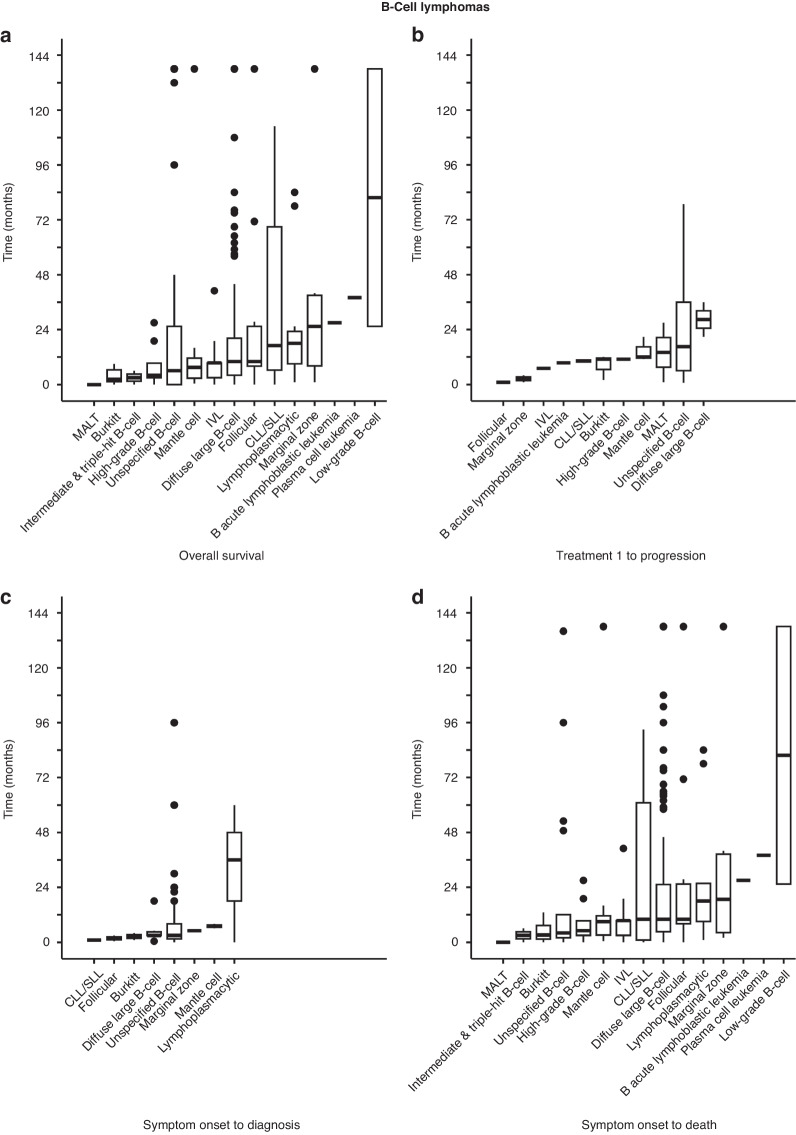
Kruskal-Wallis analysis of survival according to histological subtypes of B-cell lymphoma. **a** Analysis of OS showed a chi-squared value of 36.033 with 14 degrees of freedom and *p* = 0.01032. **b** Analysis of time from treatment 1 to progression showed a chi-squared value of 9.9899 with 10 degrees of freedom and *p* = 0.4414. **c** Analysis of time from symptom onset to diagnosis showed a chi-squared value of 4.9476 with 7 degrees of freedom and *p* = 0.6664. **d** Analysis of time from symptom onset to death showed a chi-squared value of 35.834 with 14 degrees of freedom and *p* = 0.01105. See also Supplementary Table 6A–D.

Kruskal-Wallis analysis of B-cell lymphomas revealed that OS was different among various histologic subtypes (*p* = 0.01032, Fig. 5a), particularly between low-grade B-cell lymphoma and (i) Burkitt’s lymphoma (*p* = 0.00219) and (ii) unspecified B-cell lymphoma (*p* = 0.01833) (Supplementary Table 6A). Additional analyses did not show significance for time from treatment 1 to progression (*p* = 0.4414, Fig. 5b) and time from symptom onset to diagnosis (*p* = 0.6664, Fig. 5c), but time from symptom onset to death was significant (*p* = 0.01105, Fig. 5d). Specifically, there was a significant difference between low-grade B-cell lymphoma and (i) Burkitt’s lymphoma (*p* = 0.00136), (ii) unspecified B-cell lymphoma (*p* = 0.01989), (iii) high-grade B-cell lymphoma (*p* = 0.03942) (Supplementary Table 6D). Collectively, there is a clearcut survival difference between low-grade and other types of lymphomas in NL.

We next performed separately a Kruskal-Wallis analysis on T-cell NL. There was no significant difference in OS (*p* = 0.2829), treatment 1 to progression (0.6703), time from symptom to diagnosis (*p* = 0.5847), and time from symptom onset to death (*p* = 0.2915) (Supplementary Fig. 2 and Supplementary Table 7).

**Fig. 6:**
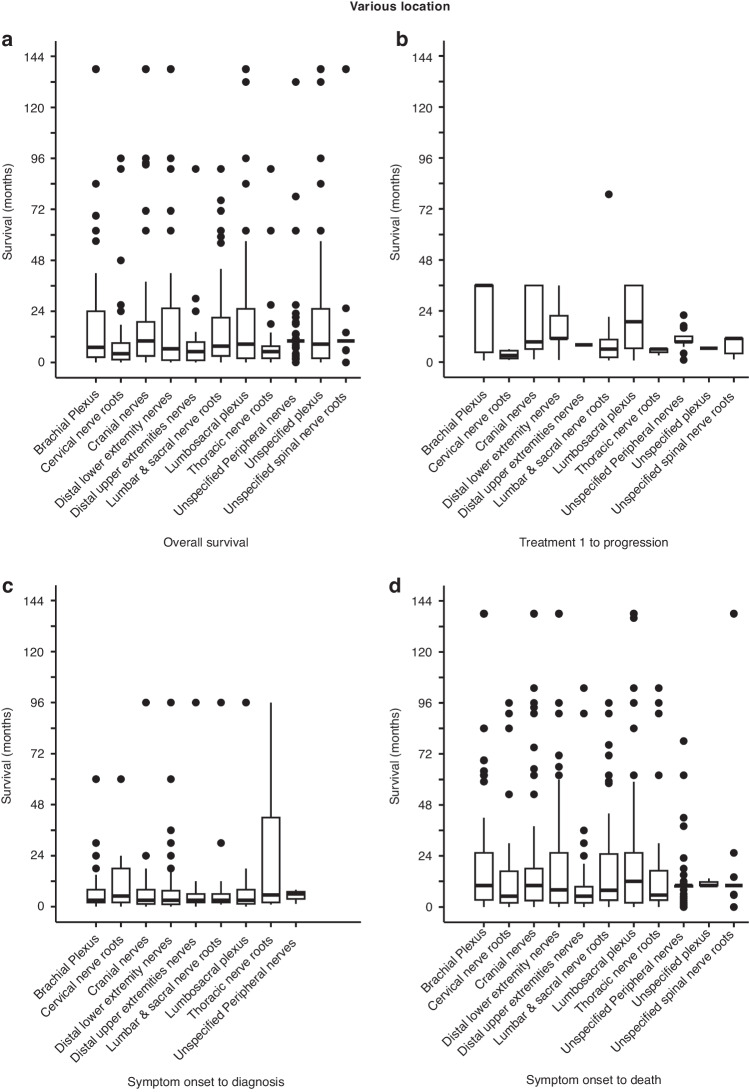
Kruskal-Wallis analysis of survival according to peripheral nerve involvement. **a** Analysis of OS showed a chi-squared value of 49.824 with 10 degrees of freedom and *p* = 0.00602. **b** Analysis of time from treatment 1 to progression showed a chi-squared value of 21.558 with 10 degrees of freedom and *p* = 0.01572. **c** Analysis of time from symptom onset to diagnosis showed a chi-squared value of 2.9153 with 10 degrees of freedom and *p* = 0.9396. **d** Analysis of time from symptom onset to death showed a chi-squared value of 33.352 with 10 degrees of freedom and *p* = 0.1534. See also Supplementary Table 8A–D.

We also examined the location of the peripheral nerves where NL first developed and asked whether the symptomatic location has prognostic significance. The most common site is brachial plexus (*n* = 142), followed by unspecified cranial nerves (*n* = 128), and sciatic nerve (*n* = 125) (Supplementary Table 3). We then performed Kruskal-Wallis analyses on various locations and noted significant variance in OS (*p* = 0.00602, Fig. 6a) and time from treatment 1 to progression (*p* = 0.01572, Fig. 6b). Locations associated with (i) the shortest OS were cervical nerve roots and nerves in distal upper extremity (Supplementary Table 8A) and (ii) the longest time from treatment 1 to progression were brachial and lumbosacral plexus (Supplementary Table 8B). No significant variance was seen in time from symptom onset to diagnosis (*p* = 0.9396, Fig. 6c and Supplementary Table 8C) and time from symptom onset to death (*p* = 0.1534, Fig. 6d and Supplementary Table 8D). Collectively, these data suggest that patients with brachial or lumbosacral plexus involvement may have a better prognosis, and this is probably secondary to a more robust blood supply that helps medical or radiation therapy.

## Discussion

NL is a rare manifestation of extranodal lymphoma and this rarity precludes prospective examination of its natural history. Although case reports and small case series have inherent publication bias and data heterogeneity, a meta-analysis of existing literature is still the only means of a comprehensive analysis of this disorder. Our literature analysis of 559 NL patients from 286 articles revealed that they had a shorter survival (median 12 months) compared to other extranodal lymphomas involving the nervous system, most notably primary central nervous system lymphoma (median 25 to >71 months) and intravascular lymphomatosis (median 19 months) [12–15]. This is most likely due to difficulties in establishing timely diagnosis so that appropriate treatment can be applied. Indeed, time from symptom onset to diagnosis did not improve over successive decades from 1950 to 2022, and the number of reported diagnoses established at autopsy did not decrease but actually increased during this period. Less than 50% (267 of 559) of our cohort had a confirmed tissue diagnosis by direct biopsy of a peripheral nerve, while the rest had the diagnosis from autopsy or tests showing respectively thickened or FDG-avid peripheral nerves on MRI or PET. Although histologic confirmation of lymphoma cells is the gold standard, it is often not feasible due to potential irreversible damage to the peripheral nerve upon biopsy [16]. Furthermore, a small number of patients (7%) underwent electromyography and nerve conduction studies. As a result, oncologists are likely forced to make a treatment decision with incomplete diagnostic information. Only 62 out of 559 (11%) patients received second treatments and 13 (2%) received three or more regimens, and these patients probably had better prognostic features than the rest of the population. Still, more accurate and timely diagnosis is prerequisite for successful treatment and this could potentially extend the dismal survival of NL patients.

NL is one of few extranodal non-Hodgkins’s lymphomas involving the nervous system. First, the most common one is primary central nervous system lymphoma (PCNSL), which is found in the brain as a solitary, homogeneously enhancing mass with infiltration along the white matter into the adjacent parenchyma. Diagnosis is usually established by stereotaxic brain biopsy, and the histology typically consists of CD20+ diffuse large B cells and they are classified as a non-germinal center subtype based on gene expression profiling and immunohistochemistry positive for CD10, Bcl-6, and MUM1 [17]. Although resection of the mass could be performed and this approach may increase patient survival, definitive benefit will most likely require confirmation in a randomized clinical [18–20]. This is because PCNSL is very chemo-sensitive and the typical patient usually achieves complete response from HD-MTX treatment [21–24]. Second, intravascular lymphomatosis is an even rarer type of extra-nodal lymphoma involving the central nervous system [15]. Although the lymphoma cells are located within the Virchow-Robin space of the cerebral vasculature and therefore on the systemic side of cerebral circulation, treatment also involves HD-MTX [25]. The molecular genetics is less certain but defects in beta-1 integrin and ICAM-1 that disabled cellular diapedesis have been identified [26]. Unfortunately, detailed immunohistochemical profiling and genetic data for NL are unknown and future work should focus on defining the immunophenotype and genotype of this malignancy.

Our work has several limitations. First, our analysis was based on heterogeneous data extracted from case reports, small patient series, and retrospective analysis from patient databases. The rarity of NL therefore precludes prospective examination of its natural history and meta-analysis is the only means of a comprehensive analysis of this disorder. This approach is similar to our prior analyses of extracranial glioblastomas and intravascular lymphomatosis, both of which are rare CNS malignancies [15, 27]. Although imperfect, our prior work on extracranial glioblastoma has provided a foundation for the current molecular characterization of these tumor cells using next generation sequencing platforms from MSK-IMPACT and Sequenom [28]. Second, there were 3 larger series of NL consisting of 50 patients from an international collaborative group as well as 40 and 25 patients from two single-institution series [4, 5, 29]. We chose to deconvolute the aggregate statistics into individual patient data rather than treating each publication as an individual data point to develop a more comparable dataset. Third, peripheral nerves can be infiltrated by leukemia cells as in neuroleukemiosis [30, 31]. As part of our global analysis of NL, we decided to be inclusive of the few cases of lymphoma- or lymphoid-like leukemic NL, which were difficult to differentiate them from lymphoma or leukemia. Finally, the optimal diagnostic modality for NL is unclear, and we did not include cases that only reported neuroimaging or neuropathology findings without any clinical data from patients. Despite these limitations, our approach most likely established a representative set of NL patients for an aggregate analysis of survival and prognostication.

In summary, it is difficult to diagnose NL and our analysis showed that timely diagnosis of this malignancy remains a challenge unchanged in past decades. A large number of patients with NL is still diagnosed post-mortem. Among those fortunate to be diagnosed pre-mortem, there has been progressive lengthening of OS and time from treatment 1 to progression. Therefore, future research effort that focuses on the diagnostic rather than treatment aspect for this malignancy will probably have a greater impact on patient survival.

## Supplementary information

Supplementary Figures

Supplementary Tables

## Data Availability

All data generated are in the supplementary figures and tables.
