# Supplementary material for: The natural history of neurolymphomatosis
Source: BJC Rep. 2024 Apr 23;2:34. doi: 10.1038/s44276-024-00053-x (PMC11523968; doi:10.1038/s44276-024-00053-x)
Supplement: Supplementary file 1 — Supplementary Figures [file 44276_2024_53_MOESM1_ESM.pdf]

Supplementary Figure 1

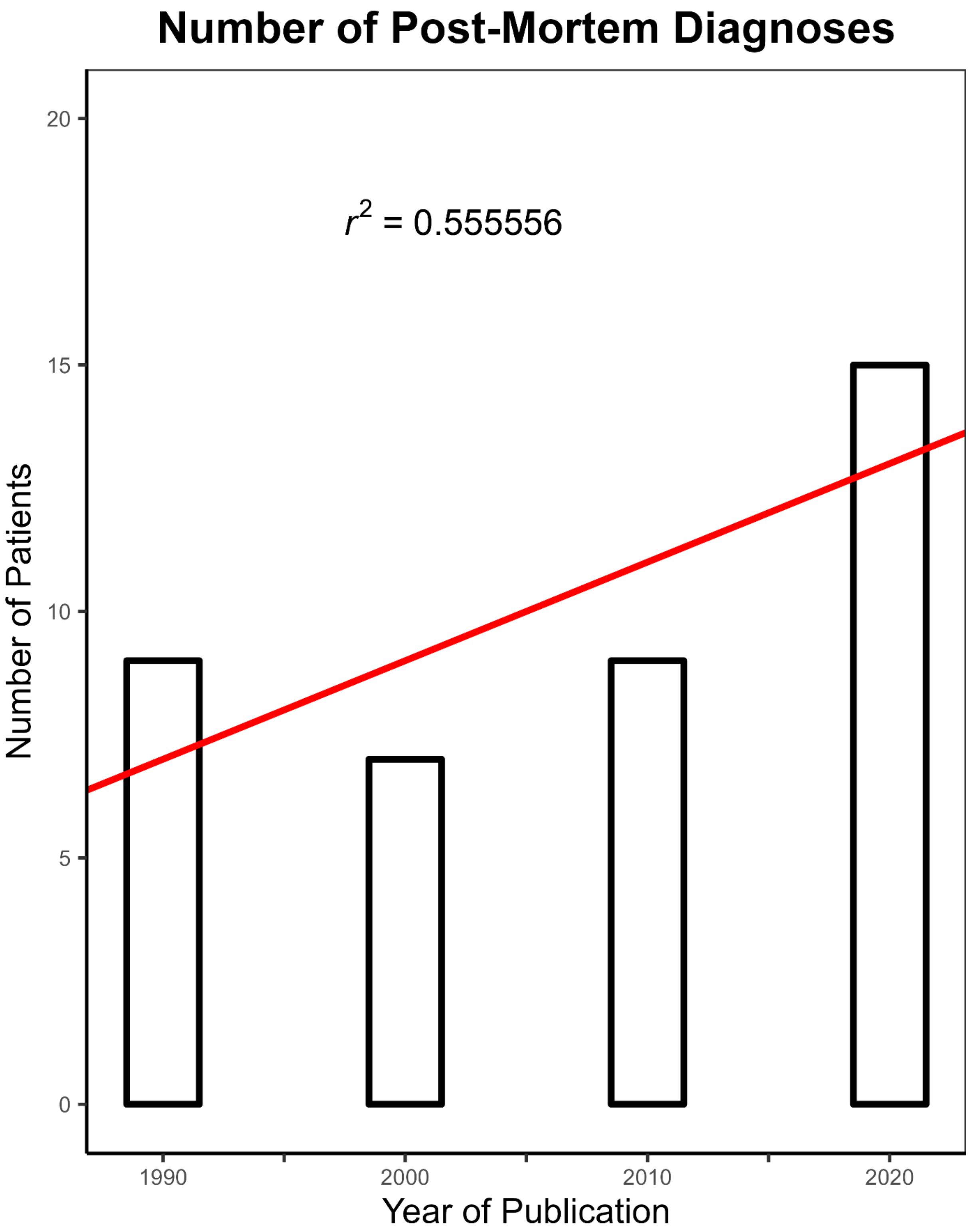

Supplemental Figure 1: Regression of the number of post-mortem diagnoses according to year of publication.

Supplementary Figure 2

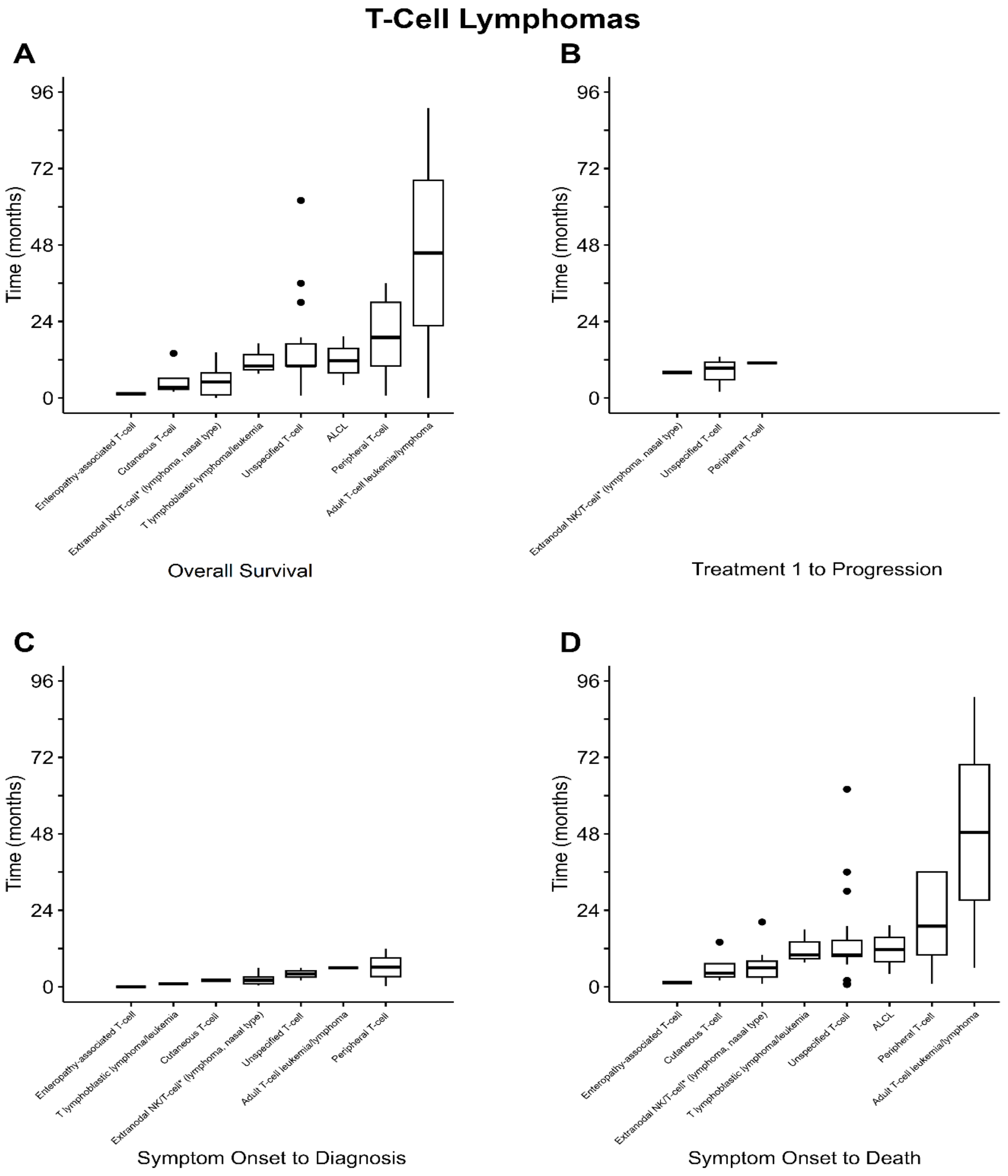

Supplemental Figure 2: Kruskal-Wallis analysis of survival according to histological subtypes of T-cell lymphoma. (A) Analysis of OS showed a chi-squared value of 8.5973 with 7 degrees of freedom and p=0.2829. (B) Analysis of time from treatment 1 to progression showed a chi-squared value of 0.8 with 2 degrees of freedom and p=0.6703. (C) Analysis of time from symptom onset to diagnosis showed a chi-squared value of 4.6854 with 6 degrees of freedom and p=0.5847. (D) Analysis of time from symptom onset to death showed a chi-squared value of 8.489 with 7 degrees of freedom and p=0.2915. See also Supplemental Table 7A-D.
