# Supplementary material for: The natural history of neurolymphomatosis
Source: BJC Rep. 2024 Apr 23;2:34. doi: 10.1038/s44276-024-00053-x (PMC11523968; doi:10.1038/s44276-024-00053-x)
Supplement: Supplementary file 2 — Supplementary Tables [file 44276_2024_53_MOESM2_ESM.pdf]

Supplemental Table 1: Citations of articles included in chronological order (n=231).

1. Radnai B, Takacs-Nagy L. [Neurolymphomatosis in man]. Orv Hetil 1951;92(48):1559-62
2. Borit A, Altrocchi PH. Recurrent polyneuropathy and neurolymphomatosis. Arch Neurol 1971;24(1):40-9 doi: 10.1001/archneur.1971.00480310068006[published Online First: Epub Date]|.
3. Trelles JO, Urquiaga C, Palomino L, Trelles L. [Human peripheral neurolymphomatosis (reticuloendothelial polyradiculoneuritis)]. J Neurol Sci 1976;28(2):187-202 doi: 10.1016/0022-510x(76)90102-7[published Online First: Epub Date]|.
4. Guberman A, Rosenbaum H, Braciale T, Schlaepfer WW. Human neurolymphomatosis. J Neurol Sci 1978;36(1):1-12 doi: 10.1016/0022-510x(78)90157-0[published Online First: Epub Date]|.
5. Trelles JO, Trelles L. [Human peripheral neurolymphomatosis]. Rev Neurol (Paris) 1983;139(12):703-14
6. Guberman A. Fatal peripheral neurolymphomatosis. Neurology 1984;34(2):259-60 doi: 10.1212/wnl.34.2.259-a[published Online First: Epub Date]|.
7. Woimant F, Schanen A, Bertail MA, et al. [Myelopathy manifesting as macroglobulinemia]. Ann Med Interne (Paris) 1985;136(2):121-4
8. Kuroda Y, Nakata H, Kakigi R, Oda K, Shibasaki H, Nakashiro H. Human neurolymphomatosis by adult T-cell leukemia. Neurology 1989;39(1):144-6 doi: 10.1212/wnl.39.1.144[published Online First: Epub Date]|.
9. Grisold W, Jellinger K, Lutz D. Human neurolymphomatosis in a patient with chronic lymphatic leukemia. Clin Neuropathol 1990;9(5):224-30
10. Diaz-Arrastia R, Younger DS, Hair L, et al. Neurolymphomatosis: a clinicopathologic syndrome re-emerges. Neurology 1992;42(6):1136-41 doi: 10.1212/wnl.42.6.1136[published Online First: Epub Date]|.
11. van den Bent MJ, de Bruin HG, Beun GD, Vecht CJ. Neurolymphomatosis of the median nerve. Neurology 1995;45(7):1403-5 doi: 10.1212/wnl.45.7.1403[published Online First: Epub Date]|.
12. Swarnkar A, Fukui MB, Fink DJ, Rao GR. MR imaging of brachial plexopathy in neurolymphomatosis. AJR Am J Roentgenol 1997;169(4):1189-90 doi: 10.2214/ajr.169.4.9308489[published Online First: Epub Date]|.
13. Abad S, Zagdanski AM, Brechignac S, Thioliere B, Brouet JC, Mariette X. Neurolymphomatosis in Waldenstrom's macroglobulinaemia. Br J Haematol 1999;106(1):100-3 doi: 10.1046/j.1365-2141.1999.01482.x[published Online First: Epub Date]|.
14. Bower SP, McKelvie P, Peppard RW, Roberts L. Neurolymphomatosis presenting as mononeuritis multiplex. J Clin Neurosci 1999;6(6):530-2 doi: 10.1016/s0967-5868(99)90020-6[published Online First: Epub Date]|.
15. van den Bent MJ, de Bruin HG, Bos GM, Brutel de la Riviere G, Sillevis Smitt PA. Negative sural nerve biopsy in neurolymphomatosis. J Neurol 1999;246(12):1159-63 doi: 10.1007/s004150050535[published Online First: Epub Date]|.
16. Carey MJ, Smith AG, Townsend JJ. Pathologic quiz case: progressive diffuse weakness after chemotherapy for large cell lymphoma in a middle-aged woman. Lymphomatous meningitis with neurolymphomatosis. Arch Pathol Lab Med 2000;124(4):645-6 doi: 10.5858/2000-124-0645-PQC[published Online First: Epub Date]|.

17. Kuntzer T, Lobrinus JA, Janzer RC, Ghika J, Bogousslavsky J. Clinicopathological and molecular biological studies in a patient with neurolymphomatosis. Muscle Nerve 2000;23(10):1604-9 doi: 10.1002/1097-4598(200010)23:10<1604::aid-mus21>3.0.co;2-f[published Online First: Epub Date]|.
18. Pietrangeli A, Milella M, De Marco S, et al. Brachial plexus neuropathy as unusual onset of diffuse neurolymphomatosis. Neurol Sci 2000;21(4):241-5 doi: 10.1007/s100720070083[published Online First: Epub Date]|.
19. Quinones-Hinojosa A, Friedlander RM, Boyer PJ, Batchelor TT, Chiocca EA. Solitary sciatic nerve lymphoma as an initial manifestation of diffuse neurolymphomatosis. Case report and review of the literature. J Neurosurg 2000;92(1):165-9 doi: 10.3171/jns.2000.92.1.0165[published Online First: Epub Date]|.
20. Moore KR, Blumenthal DT, Smith AG, Ward JH. Neurolymphomatosis of the lumbar plexus: high-resolution MR neurography findings. Neurology 2001;57(4):740-2 doi: 10.1212/wnl.57.4.740[published Online First: Epub Date]|.
21. Odabasi Z, Parrott JH, Reddy VV, Oh SJ. Neurolymphomatosis associated with muscle and cerebral involvement caused by natural killer cell lymphoma: a case report and review of literature. J Peripher Nerv Syst 2001;6(4):197-203 doi: 10.1046/j.1529-8027.2001.01018.x[published Online First: Epub Date]|.
22. Trojan A, Jermann M, Taverna C, Hany TF. Fusion PET-CT imaging of neurolymphomatosis. Ann Oncol 2002;13(5):802-5 doi: 10.1093/annonc/mdf057[published Online First: Epub Date]|.
23. Baehring JM, Damek D, Martin EC, Betensky RA, Hochberg FH. Neurolymphomatosis. Neuro Oncol 2003;5(2):104-15 doi: 10.1093/neuonc/nop021[published Online First: Epub Date]|.
24. Dakwar E, Teja S, Alleyne CH, Jr. Sciatic neurolymphomatosis. Neurology 2004;63(9):1751 doi: 10.1212/01.wnl.0000138501.06028.68[published Online First: Epub Date]|.
25. Ghobrial IM, Buadi F, Spinner RJ, et al. High-dose intravenous methotrexate followed by autologous stem cell transplantation as a potentially effective therapy for neurolymphomatosis. Cancer 2004;100(11):2403-7 doi: 10.1002/cncr.20263[published Online First: Epub Date]|.
26. Pages M, Marty-Double C, Pages AM. Sensory neuropathy as revealing symptom of neurolymphomatosis: report of a case with a 15-year duration. Eur Neurol 2004;52(1):57-8 doi: 10.1159/000079545[published Online First: Epub Date]|.
27. Bi HY, Na J, Zang GM, Zhang W, Yuan Y. [Neurolymphomatosis, a case report]. Beijing Da Xue Xue Bao Yi Xue Ban 2005;37(3):331
28. Bokstein F, Goor O, Shihman B, et al. Assessment of neurolymphomatosis by brachial plexus biopsy and PET/CT. Report of a case. J Neurooncol 2005;72(2):163-7 doi: 10.1007/s11060-004-3389-0[published Online First: Epub Date]|.
29. Kanter P, Zeidman A, Streifler J, et al. PET-CT imaging of combined brachial and lumbosacral neurolymphomatosis. Eur J Haematol 2005;74(1):66-9 doi: 10.1111/j.1600-0609.2004.00369.x[published Online First: Epub Date]|.
30. Kim JH, Jang JH, Koh SB. A case of neurolymphomatosis involving cranial nerves: MRI and fusion PET-CT findings. J Neurooncol 2006;80(2):209-10 doi: 10.1007/s11060-006-9164-7[published Online First: Epub Date]|.
31. Ozturk E, Arpaci F, Kocaoglu M, Arslan N, Bulakbasi N, Ozguven M. Detection of widespread neurolymphomatosis with 18F-FDG PET. Eur J Nucl Med Mol Imaging 2006;33(8):975-6 doi: 10.1007/s00259-006-0146-y[published Online First: Epub Date]|.

32. Rosso SM, de Bruin HG, Wu KL, van den Bent MJ. Diagnosis of neurolymphomatosis with FDG PET. Neurology 2006;67(4):722-3 doi: 10.1212/01.wnl.0000230155.67464.37[published Online First: Epub Date]|.
33. Strobel K, Pestalozzi B, Ciernik I, Schaefer NG, Koma AY, Hany TF. Changing PET/CT manifestation of neurolymphomatosis. Eur J Nucl Med Mol Imaging 2006;33(10):1244 doi: 10.1007/s00259-006-0112-8[published Online First: Epub Date]|.
34. Grisold W, Klimpfinger M, Maehr B, et al. Peripheral nerve involvement in lymphoma: the meninges as the crucial barrier between meningoradicular spread and neurolymphomatosis. J Peripher Nerv Syst 2007;12(1):58-60 doi: 10.1111/j.1529-8027.2007.00120.x[published Online First: Epub Date]|.
35. Hoshikawa Y, Oguri T, Hattori M, et al. [A case of neurolymphomatosis diagnosed with FDG-PET]. Rinsho Shinkeigaku 2007;47(7):437-40
36. Rahmani M, Birouk N, Amarti A, et al. [T-cell lymphoma revealed by a mononeuritis multiplex: case report and review of literature]. Rev Neurol (Paris) 2007;163(4):462-70 doi: 10.1016/s0035-3787(07)90422-1[published Online First: Epub Date]|.
37. Shibata-Hamaguchi A, Samuraki M, Furui E, et al. B-cell neurolymphomatosis confined to the peripheral nervous system. J Neurol Sci 2007;260(1-2):249-52 doi: 10.1016/j.jns.2007.03.029[published Online First: Epub Date]|.
38. Strobel K, Fischer K, Hany TF, Poryazova R, Jung HH. Sciatic nerve neurolymphomatosis - extent and therapy response assessment with PET/CT. Clin Nucl Med 2007;32(8):646-8 doi: 10.1097/RLU.0b013e3180a1ac74[published Online First: Epub Date]|.
39. Tachibana T, Tomita N, Ueda T, et al. [Systemic neurolymphomatosis complicated in diffuse large B-cell lymphoma]. Rinsho Ketsueki 2007;48(12):1563-6
40. Yazawa S, Ohi T, Shiomi K, Takashima N, Kyoraku I, Nakazato M. Brachial plexus neurolymphomatosis: a discrepancy between electrophysiological and radiological findings. Intern Med 2007;46(8):533-4 doi: 10.2169/internalmedicine.46.6153[published Online First: Epub Date]|.
41. Chaturvedi A, Singh JP, Rastogi V. MRI diagnosis of neurolymphomatosis of the brachial plexus. Neurol India 2008;56(4):485-6 doi: 10.4103/0028-3886.44817[published Online First: Epub Date]|.
42. Czepczynski R, Guzikowska-Ruszkowska I, Sowinski J. Neurolymphomatosis detected by (18)F-FDG PET/CT scan - a case report. Nucl Med Rev Cent East Eur 2008;11(2):73-5
43. Dong Q, Wong KK, Avram AM. Sacral nerve root neurolymphomatosis diagnosed on FDG-PET/CT and magnetic resonance imaging. Clin Nucl Med 2008;33(1):30-1 doi: 10.1097/RLU.0b013e31815c5020[published Online First: Epub Date]|.
44. Khong P, Pitham T, Owler B. Isolated neurolymphomatosis of the cauda equina and filum terminale: case report. Spine (Phila Pa 1976) 2008;33(21):E807-11 doi: 10.1097/BRS.0b013e31818441be[published Online First: Epub Date]|.
45. Levin N, Soffer D, Grissaru S, Aizikovich N, Gomori JM, Siegal T. Primary T-cell CNS lymphoma presenting with leptomeningeal spread and neurolymphomatosis. J Neurooncol 2008;90(1):77-83 doi: 10.1007/s11060-008-9633-2[published Online First: Epub Date]|.
46. Lin M, Kilanowska J, Taper J, Chu J. Neurolymphomatosis--diagnosis and assessment of treatment response by FDG PET-CT. Hematol Oncol 2008;26(1):43-5 doi: 10.1002/hon.837[published Online First: Epub Date]|.

47. Shima K, Ishida C, Okino S, Kotani T, Higashi K, Yamada M. A linear lesion along the brachial plexus on FDG-PET in neurolymphomatosis. Intern Med 2008;47(12):1159-60 doi: 10.2169/internalmedicine.47.1104[published Online First: Epub Date]|.
48. Urban PP, Kaczmarek E, Wellach I, et al. [Neurolymphomatosis. Subacute sensorimotor polyneuropathy as a first sign of non-Hodgkin's B cell lymphoma]. Nervenarzt 2008;79(6):699-702 doi: 10.1007/s00115-008-2456-8[published Online First: Epub Date]|.
49. Bezier M, Reguiai Z, Delaby P, et al. Neurolymphomatosis associated with Sezary syndrome. Arch Dermatol 2009;145(3):294-6 doi: 10.1001/archdermatol.2008.584[published Online First: Epub Date]|.
50. Chamberlain MC, Fink J. Neurolymphomatosis: a rare metastatic complication of diffuse large B-Cell lymphoma. J Neurooncol 2009;95(2):285-88 doi: 10.1007/s11060-009-9918-0[published Online First: Epub Date]|.
51. Duran C, Infante JR, Serrano J, et al. [Neurolymphomatosis: diagnosis of extension and assessment of response to treatment with PET-CT]. Rev Esp Med Nucl 2009;28(6):295-8 doi: 10.1016/j.remn.2009.07.002[published Online First: Epub Date]|.
52. Hong RS, Woodson EA, Hansen MR. Neurolymphomatosis mimicking chemotherapy-induced ototoxicity. Otol Neurotol 2009;30(4):566-9 doi: 10.1097/MAo.0b013e3181a527b2[published Online First: Epub Date]|.
53. Kosa SC, Peller PJ, Klein CJ. T-cell neurolymphomatosis involving cauda equina and sciatic nerves. Neurology 2009;72(1):98 doi: 10.1212/01.wnl.0000338598.07063.5b[published Online First: Epub Date]|.
54. Nishio M, Tamaki T, Ochi H, Shibamoto Y. Intraspinal canal neurolymphomatosis detected by FDG-PET/CT. Clin Nucl Med 2009;34(9):610-2 doi: 10.1097/RLU.0b013e3181b06c76[published Online First: Epub Date]|.
55. Peruzzi P, Ray-Chaudhuri A, Slone WH, Mekhjian HS, Porcu P, Chiocca E. Reversal of neurological deficit after chemotherapy in BCL-6-positive neurolymphomatosis. Case report. J Neurosurg 2009;111(2):247-51 doi: 10.3171/2008.11.JNS08291[published Online First: Epub Date]|.
56. von Falck C, Rodt T, Joerdens S, et al. F-18 2-fluoro-2-deoxy-glucose positron emission tomography/computed tomography for the detection of radicular and peripheral neurolymphomatosis: correlation with magnetic resonance imaging and ultrasound. Clin Nucl Med 2009;34(8):493-5 doi: 10.1097/RLU.0b013e3181abb6ba[published Online First: Epub Date]|.
57. Gan HK, Azad A, Cher L, Mitchell PL. Neurolymphomatosis: diagnosis, management, and outcomes in patients treated with rituximab. Neuro Oncol 2010;12(2):212-5 doi: 10.1093/neuonc/nop021[published Online First: Epub Date]|.
58. Grisariu S, Avni B, Batchelor TT, et al. Neurolymphomatosis: an International Primary CNS Lymphoma Collaborative Group report. Blood 2010;115(24):5005-11 doi: 10.1182/blood-2009-12-258210[published Online First: Epub Date]|.
59. Koyama T, O'Uchi T, Matsue K. Neurolymphomatosis involving the trigeminal nerve and deep peroneal nerve in a patient with relapsed intravascular large B-cell lymphoma. Eur J Haematol 2010;85(3):275-6 doi: 10.1111/j.1600-0609.2010.01477.x[published Online First: Epub Date]|.
60. Miki M, Masaki Y, Nakamura T, et al. [Primary neurolymphomatosis of the cervical nerve root]. Rinsho Ketsueki 2010;51(7):564-7

61. Nishizawa M, Yamashita K, Nakamoto Y, Kotani S, Kondo T, Takaori-Kondo A. Neurolymphomatosis as a manifestation of relapsed primary cardiac lymphoma. Int J Hematol 2010;92(5):679-80 doi: 10.1007/s12185-010-0716-4[published Online First: Epub Date]|.
62. Santos E, Scolding NJ. Neurolymphomatosis mimicking neurosarcoidosis: a case report. J Med Case Rep 2010;4:5 doi: 10.1186/1752-1947-4-5[published Online First: Epub Date]|.
63. Ye BS, Sunwoo IN, Suh BC, Park JP, Shim DS, Kim SM. Diffuse large B-cell lymphoma presenting as piriformis syndrome. Muscle Nerve 2010;41(3):419-22 doi: 10.1002/mus.21538[published Online First: Epub Date]|.
64. Cassereau J, Letournel F, Francois S, Dubas F, Nicolas G. Chronic inflammatory demyelinating polyneuropathy in Waldenstrom's macroglobulinemia. Rev Neurol (Paris) 2011;167(4):343-7 doi: 10.1016/j.neurol.2010.10.015[published Online First: Epub Date]|.
65. Hong CM, Lee SW, Lee HJ, et al. Neurolymphomatosis on F-18 FDG PET/CT and MRI Findings: A Case Report. Nucl Med Mol Imaging 2011;45(1):76-8 doi: 10.1007/s13139-010-0070-8[published Online First: Epub Date]|.
66. Hung MH, Gau JP. Relapsed testicular lymphoma presenting with cranial nerve neurolymphomatosis. Neurology 2011;76(16):1441 doi: 10.1212/WNL.0b013e318216711a[published Online First: Epub Date]|.
67. Matsue K, Hayama BY, Iwama K, et al. High frequency of neurolymphomatosis as a relapse disease of intravascular large B-cell lymphoma. Cancer 2011;117(19):4512-21 doi: 10.1002/cncr.26090[published Online First: Epub Date]|.
68. Suga K, Yasuhiko K, Matsunaga N, Yujiri T, Nakazora T, Ariyoshi K. F-18 FDG PET/CT findings of a case of sacral nerve root neurolymphomatosis that occurred during chemotherapy. Clin Nucl Med 2011;36(1):73-6 doi: 10.1097/RLU.0b013e3181f4a03f[published Online First: Epub Date]|.
69. Talanow R, Shrikanthan S. Value of FDG PET in the evaluation of therapy response in nerve root neurolymphomatosis. Clin Nucl Med 2011;36(5):389-91 doi: 10.1097/RLU.0b013e31820a9fab[published Online First: Epub Date]|.
70. Casselberry JN, Kritz AD. Neurolymphomatosis: a case study of diffuse large B-cell lymphoma. Clin J Oncol Nurs 2012;16(6):636-7 doi: 10.1188/12.CJON.636-637[published Online First: Epub Date]|.
71. Cheung C, Lopes D, Hung KN, Chan T, Chan KW, Kwong YL. Neurolymphomatosis: role of positron emission tomography in diagnosis. Ann Hematol 2012;91(8):1313-4 doi: 10.1007/s00277-011-1379-9[published Online First: Epub Date]|.
72. D'Amico A, Napoli M, Cirillo M, et al. Imaging of cervical extradural en-plaque meningioma. A case report. Neuroradiol J 2012;25(5):598-603 doi: 10.1177/197140091202500514[published Online First: Epub Date]|.
73. Gs B, J G, S LAM, et al. Bilateral vagus nerve neurolymphomatosis diagnosed using PET/CT and diffusion-weighted MRI. Clin Nucl Med 2012;37(9):e225-8 doi: 10.1097/RLU.0b013e318251e474[published Online First: Epub Date]|.
74. Harada N, Tomita M, Kimura A, et al. [Case report; Two cases of neurolymphomatosis as a manifestation of relapsed non-Hodgkin lymphoma]. Nihon Naika Gakkai Zasshi 2012;101(1):157-60 doi: 10.2169/naika.101.157[published Online First: Epub Date]|.
75. Pentsova E, Rosenblum M, Holodny A, Palomba ML, Omuro A. Chemotherapy-related magnetic resonance imaging abnormalities mimicking disease progression following intraventricular

liposomal cytarabine and high dose methotrexate for neurolymphomatosis. Leuk Lymphoma 2012;53(8):1620-2 doi: 10.3109/10428194.2012.656632[published Online First: Epub Date]|.
76. Sugai A, Konno T, Yano T, Umeda M, Oyake M, Fujita N. [Neurolymphomatosis presenting as bilateral tongue atrophy: a case report]. Rinsho Shinkeigaku 2012;52(8):589-91 doi: 10.5692/clinicalneurol.52.589[published Online First: Epub Date]|.
77. Tsang HH, Lee EY, Anthony MP, Khong PL. 18F-FDG PET/CT diagnosis of vagus nerve neurolymphomatosis. Clin Nucl Med 2012;37(9):897-8 doi: 10.1097/RLU.0b013e31825b2042[published Online First: Epub Date]|.
78. Vecchio D, Mittino D, Terazzi E, Nassi L, Conconi A, Monaco F. A case of cranial multinevritis: from the onset to the diagnosis of primary neurolymphomatosis. BMJ Case Rep 2012;2012 doi: 10.1136/bcr.06.2011.4299[published Online First: Epub Date]|.
79. Wan MY, Ardeshna KM, Bomanji J. Neurolymphomatosis in a patient with lymphoblastic lymphoma. Br J Haematol 2012;156(6):691 doi: 10.1111/j.1365-2141.2011.09002.x[published Online First: Epub Date]|.
80. Xu L, Zhou Y, Qiu D, Shams S. Fusion PET-CT detection of neurolymphomatosis originating from primary breast lymphoma: A case report and literature review. Oncol Lett 2012;4(5):973-75 doi: 10.3892/ol.2012.840[published Online First: Epub Date]|.
81. Yamada S, Tanimoto A, Nabeshima A, et al. Diffuse large B-cell lymphoma presenting with neurolymphomatosis and intravascular lymphoma: a unique autopsy case with diverse neurological symptoms. Diagn Pathol 2012;7:94 doi: 10.1186/1746-1596-7-94[published Online First: Epub Date]|.
82. Blioskas S, Tsaligopoulos M, Kyriafinis G, et al. Bilateral secondary neurolymphomatosis of the internal auditory canal nerves: a case report. Am J Otolaryngol 2013;34(5):556-8 doi: 10.1016/j.amjoto.2013.04.002[published Online First: Epub Date]|.
83. Choi YJ, Shin JA, Kim YH, et al. Neurolymphomatosis of Brachial Plexus in Patients with Non-Hodgkin's Lymphoma. Case Rep Oncol Med 2013;2013:492329 doi: 10.1155/2013/492329[published Online First: Epub Date]|.
84. Hanna R, Di Primio GA, Schweitzer M, Torres C, Sheikh A, Chakraborty S. Progressive neurolymphomatosis with cutaneous disease: response in a patient with mycosis fungoides. Skeletal Radiol 2013;42(7):1011-5 doi: 10.1007/s00256-013-1595-6[published Online First: Epub Date]|.
85. Okada M, Takamatsu K, Oebisu N, Nakamura H. Solitary neurolymphomatosis of the brachial plexus mimicking benign nerve sheath tumour: case report. Br J Neurosurg 2013;27(3):386-7 doi: 10.3109/02688697.2012.737959[published Online First: Epub Date]|.
86. Ramirez-Zamora A, Morales-Vidal S, Chawla J, Biller J. Autopsy proven peripheral nervous system neurolymphomatosis despite negative bilateral sural nerve biopsy. Front Neurol 2013;4:197 doi: 10.3389/fneur.2013.00197[published Online First: Epub Date]|.
87. Salm LP, Van der Hiel B, Stokkel MP. Neurolymphomatosis diagnosed by (18)F-FDG PET-CT. Clin Nucl Med 2013;38(6):e261-2 doi: 10.1097/RLU.0b013e318266ce70[published Online First: Epub Date]|.
88. Tanaka H, Yoshino K, Sakaida E, et al. Secondary neurolymphomatosis detected by whole-body diffusion-weighted magnetic resonance imaging: a case report. J Clin Exp Hematop 2013;53(3):221-6 doi: 10.3960/jslrt.53.221[published Online First: Epub Date]|.

89. Toledano M, Siddiqui MA, Thompson CA, Garza I, Pittock SJ. Teaching NeuroImages: diagnostic utility of FDG-PET in neurolymphomatosis. Neurology 2013;81(1):e3 doi: 10.1212/WNL.0b013e318297eef5[published Online First: Epub Date]|.
90. Tomita M, Koike H, Kawagashira Y, et al. Clinicopathological features of neuropathy associated with lymphoma. Brain 2013;136(Pt 8):2563-78 doi: 10.1093/brain/awt193[published Online First: Epub Date]|.
91. Akagi A, Ono K, Hamaguchi T, et al. Neurolymphomatosis exhibiting repeated exacerbation and remission in both the peripheral and central nervous systems. J Neurol Sci 2014;345(1-2):267-8 doi: 10.1016/j.jns.2014.07.038[published Online First: Epub Date]|.
92. Canh NX, Tan NV, Tung TT, Son NT, Maurea S. (18)F-FDG PET/CT in Neurolymphomatosis: Report of 3 Cases. Asia Ocean J Nucl Med Biol 2014;2(1):57-64
93. Del Grande A, Sabatelli M, Luigetti M, et al. Primary multifocal lymphoma of peripheral nervous system: case report and review of the literature. Muscle Nerve 2014;50(6):1016-22 doi: 10.1002/mus.24354[published Online First: Epub Date]|.
94. Jung YH, Woo IS, Han DJ, Han CW. (18)F-fluoro-2-deoxy-D-glucose positron emission tomography findings of neurolymphomatosis. Blood Res 2014;49(2):83 doi: 10.5045/br.2014.49.2.83[published Online First: Epub Date]|.
95. Kajary K, Molnar Z, Miko I, Barsi P, Lengyel Z, Szakall S, Jr. Neurolymphomatosis as a late relapse of non-Hodgkin's lymphoma detected by 18F-FDG PET/CT: a case report. Rev Esp Med Nucl Imagen Mol 2014;33(1):39-42 doi: 10.1016/j.remn.2013.03.004[published Online First: Epub Date]|.
96. Kamiya-Matsuoka C, Shroff S, Gildersleeve K, Hormozdi B, Manning JT, Woodman KH. Neurolymphomatosis: a case series of clinical manifestations, treatments, and outcomes. J Neurol Sci 2014;343(1-2):144-8 doi: 10.1016/j.jns.2014.05.058[published Online First: Epub Date]|.
97. Lagarde S, Tabouret E, Matta M, et al. Primary neurolymphomatosis diagnosis and treatment: a retrospective study. J Neurol Sci 2014;342(1-2):178-81 doi: 10.1016/j.jns.2014.04.030[published Online First: Epub Date]|.
98. Ogawa S, Fukunaga A, Kanata M, et al. Neurolymphomatosis associated with erythrodermic mycosis fungoides. Acta Derm Venereol 2014;94(2):227-8 doi: 10.2340/00015555-1684[published Online First: Epub Date]|.
99. Padma S, Sundaram PS, Praveen Kumar SL. Primary peripheral neurolymphomatosis mimicking synovial sarcoma: FDG PETCT to the rescue. J Cancer Res Ther 2014;10(3):726-9 doi: 10.4103/0973-1482.136038[published Online First: Epub Date]|.
100. Peterson J, Caliskan B, Bonyadlou S. Positron emission tomography/computerized tomography imaging of multiple focus of neurolymphomatosis. Indian J Nucl Med 2014;29(4):252-3 doi: 10.4103/0972-3919.142632[published Online First: Epub Date]|.
101. Ramirez Ocana D, Gutierrez Cardo AL, Gonzalez Diaz L, Hurst K, Espeso de Haro M. [Neurolymphomatosis as initial manifestation of recurrence in lymphoma]. Rev Esp Med Nucl Imagen Mol 2014;33(1):50-1 doi: 10.1016/j.remn.2013.02.003[published Online First: Epub Date]|.
102. Sakai N, Ito-Yamashita T, Takahashi G, et al. Primary neurolymphomatosis of the lower cranial nerves presenting as Dysphagia and hoarseness: a case report. J Neurol Surg Rep 2014;75(1):e62-6 doi: 10.1055/s-0033-1363505[published Online First: Epub Date]|.

103. Shimizu I, Hamano Y, Sato S, et al. Neurolymphomatosis in a patient with extranodal NK/T-cell lymphoma, nasal-type: a case report and literature review. Intern Med 2014;53(5):471-5 doi: 10.2169/internalmedicine.53.0262[published Online First: Epub Date]|.
104. Tabuchi S, Yoshioka H, Nakayasu H, Watanabe T. Primary Central Nervous System Lymphoma of the Cerebellopontine Angle That Initially Occurred as Neurolymphomatosis of the Acoustic Nerve. NMC Case Rep J 2014;1(1):28-32 doi: 10.2176/nmccrj.2013-0353[published Online First: Epub Date]|.
105. Yilmaz S, Sager S, Sen F, Halac M. Bilateral trigeminal nerve recurrence of non-hodgkin lymphoma revealed with FDG PET/CT. Indian J Nucl Med 2014;29(1):50-2 doi: 10.4103/0972-3919.125778[published Online First: Epub Date]|.
106. Zhou WL, Wu HB, Weng CS, et al. Usefulness of 18F-FDG PET/CT in the detection of neurolymphomatosis. Nucl Med Commun 2014;35(11):1107-11 doi: 10.1097/MNM.0000000000000181[published Online First: Epub Date]|.
107. Advani P, Jiang L, Srinivasan S, Foran J. Disseminated extranodal marginal zone lymphoma involving the gastrocnemius muscle with sural neurolymphomatosis. Ann Hematol 2015;94(11):1939-40 doi: 10.1007/s00277-015-2453-5[published Online First: Epub Date]|.
108. Advani P, Paulus A, Murray P, et al. A rare case of primary high-grade large B-cell lymphoma of the sciatic nerve. Clin Lymphoma Myeloma Leuk 2015;15(6):e117-20 doi: 10.1016/j.clml.2014.12.001[published Online First: Epub Date]|.
109. Biswal CK, Mittal BR, Shukla J, Vatsa R, Bhattacharya A, Prabhakar S. Fluorothymidine PET/CT in neurolymphomatosis. Clin Nucl Med 2015;40(5):e290-2 doi: 10.1097/RLU.0000000000000632[published Online First: Epub Date]|.
110. Brandstadter R, Brody J, Morgello S, et al. Primary Neurolymphomatosis Presenting With Polyradiculoneuropathy Affecting One Lower Limb. J Clin Neuromuscul Dis 2015;17(1):6-12 doi: 10.1097/CND.0000000000000088[published Online First: Epub Date]|.
111. Ebihara Y, Ishii N, Akizuki K, et al. [A case of neurolymphomatosis: peripheral neuropathy induced by diffuse large B-cell lymphoma without any abnormal accumulation observed on early positron emission tomography-computed tomography]. Brain Nerve 2015;67(2):219-23 doi: 10.11477/mf.1416200116[published Online First: Epub Date]|.
112. Gutierrez-Martinez AJ, Malo-de-Molina R, Garcia-Garcia N. [Multiple cranial neuropathy as a manifestation of primary neurolymphomatosis]. Rev Neurol 2015;61(8):381-3
113. Lahoria R, Dyck PJ, Macon WR, et al. Neurolymphomatosis: A report of 2 cases representing opposite ends of the clinical spectrum. Muscle Nerve 2015;52(3):449-54 doi: 10.1002/mus.24646[published Online First: Epub Date]|.
114. Liu Z, Jiang T, Hou N, Jia Y. Peripheral neurolymphomatosis with tracheal asphyxia: a case report and literature review. BMC Neurol 2015;15:149 doi: 10.1186/s12883-015-0405-2[published Online First: Epub Date]|.
115. Sato M, Furuta M, Hirayanagi K, Nagamine S, Makioka K, Ikeda Y. [A case of neurolymphomatosis presenting extended involvement of spinal nerve roots]. Rinsho Shinkeigaku 2015;55(5):333-8 doi: 10.5692/clinicalneurol.cn-000615[published Online First: Epub Date]|.
116. Shaikh F, Chan AC, Awan O, et al. Diagnostic Yield of FDG-PET/CT, MRI, and CSF Cytology in Non-Biopsiable Neurolymphomatosis as a Heralding Sign of Recurrent Non-Hodgkin's Lymphoma. Cureus 2015;7(9):e319 doi: 10.7759/cureus.319[published Online First: Epub Date]|.

117. Shaikh F, Savells D, Awan O, et al. Quantitative Imaging Analysis of FDG PET/CT Imaging for Detection of Central Neurolymphomatosis in a Case of Recurrent Diffuse B-Cell Lymphoma. Cureus 2015;7(11):e379 doi: 10.7759/cureus.379[published Online First: Epub Date]|.
118. Sunami Y, Gotoh A, Hamano Y, et al. Various neurological symptoms by neurolymphomatosis as the initial presentation of primary testicular lymphoma. Case Rep Oncol 2015;8(1):200-4 doi: 10.1159/000381874[published Online First: Epub Date]|.
119. Tong AK, Neo SH, Kok TY. Disseminated Lymphoma Evolving into Neurolymphomatosis during Mid-cycle of Chemotherapy Detected by (18)F-FDG PET/CT. Ann Acad Med Singap 2015;44(11):545-7
120. Tsai HH, Chen YF, Hsieh ST, Chao CC. Neurological picture. Neurolymphomatosis as the primary presentation of non-Hodgkin's Lymphoma. J Neurol Neurosurg Psychiatry 2015;86(8):929-30 doi: 10.1136/jnnp-2014-308244[published Online First: Epub Date]|.
121. Vijayan J, Chan YC, Therimadasamy A, Wilder-Smith EP. Role of combined B-mode and Doppler sonography in evaluating neurolymphomatosis. Neurology 2015;85(9):752-5 doi: 10.1212/WNL.0000000000001880[published Online First: Epub Date]|.
122. Wang Y, Wang J, Jiang C, Peng Q, Wu M, Ma X. Secondary neurolymphomatosis of spinal nerve roots detected by (1)(8)F-FDG PET/CT: a case report and differential diagnosis of the case. Hell J Nucl Med 2015;18(3):261-3 doi: 10.1967/s002449910309[published Online First: Epub Date]|.
123. Bruce D, Eagleton H, Subesinghe M. Diagnostic and response assessment FDG PET-CT in neurolymphomatosis. Clin Case Rep 2016;4(12):1172-74 doi: 10.1002/ccr3.734[published Online First: Epub Date]|.
124. de Jong A, Mous R, van Dongen GA, Hoekstra OS, Nievelstein RA, de Keizer B. (89) Zr-rituximab PET/CT to detect neurolymphomatosis. Am J Hematol 2016;91(6):649-50 doi: 10.1002/ajh.24328[published Online First: Epub Date]|.
125. Denays R, Baudrez V, Abouhamad P, Dereme T, Milbouw G, Hermanne JP. Plexiform neurolymphomatosis. Rev Neurol (Paris) 2016;172(4-5):328-30 doi: 10.1016/j.neurol.2016.02.008[published Online First: Epub Date]|.
126. Gammon B, Gammon BR, Kim YH, Kim J. Neurotropic Gamma-Delta T-Cell Lymphoma With CD30-Positive Lymphoid Infiltrates. Am J Dermatopathol 2016;38(9):e133-6 doi: 10.1097/DAD.0000000000000560[published Online First: Epub Date]|.
127. Gykiere P, Jans L, Degrieck B, Goethals I. Neurolymphomatosis on 18F-FDG PET/CT: Diagnosis and Therapy Response. Clin Nucl Med 2016;41(2):142-3 doi: 10.1097/RLU.0000000000000982[published Online First: Epub Date]|.
128. He W, Wang W, Gustas C, Malysz J, Kaur D. Isolated sciatic neuropathy as an initial manifestation of a high grade B-cell lymphoma: A case report and literature review. Clin Neurol Neurosurg 2016;149:147-53 doi: 10.1016/j.clineuro.2016.07.029[published Online First: Epub Date]|.
129. Kinoshita H, Yamakado H, Kitano T, et al. Diagnostic utility of FDG-PET in neurolymphomatosis: report of five cases. J Neurol 2016;263(9):1719-26 doi: 10.1007/s00415-016-8190-4[published Online First: Epub Date]|.
130. Lim AT, Clucas D, Khoo C, Parameswaran BK, Lau E. Neurolymphomatosis: MRI and (18) FDG-PET features. J Med Imaging Radiat Oncol 2016;60(1):92-5 doi: 10.1111/1754-9485.12321[published Online First: Epub Date]|.

131. Oner AO, Okuyucu K, Alagoz E, Battal B, Arslan N. An Extremely Rare Intersection: Neurolymphomatosis in a Patient with Burkitt Lymphoma Detected by 18F-Fluorodeoxyglucose Positron Emission Tomography/Computed Tomography. World J Nucl Med 2016;15(3):209-11 doi: 10.4103/1450-1147.172304[published Online First: Epub Date]|.
132. Pham M, Awad M. Lymphoma relapse presenting as neurolymphomatosis. Asian J Neurosurg 2016;11(1):73 doi: 10.4103/1793-5482.165783[published Online First: Epub Date]|.
133. Scharf EL, Hanson CA, Howard MT, Keegan BM. Serial cerebrospinal fluid examinations to diagnose hematological malignancy causing neurological disease. J Neurooncol 2016;129(1):77-83 doi: 10.1007/s11060-016-2140-y[published Online First: Epub Date]|.
134. Shree R, Goyal MK, Modi M, et al. The Diagnostic Dilemma of Neurolymphomatosis. J Clin Neurol 2016;12(3):274-81 doi: 10.3988/jcn.2016.12.3.274[published Online First: Epub Date]|.
135. Sideras PA, Matthews J, Sakib SM, Ofikwu F, Spektor V. Neurolymphomatosis of the peripheral nervous system: a case report and review of the literature. Clin Imaging 2016;40(6):1253-56 doi: 10.1016/j.clinimag.2016.08.014[published Online First: Epub Date]|.
136. Umeda M, Kondo T, Nishikori M, et al. A case of neurolymphomatosis caused by follicular lymphoma successfully treated with bendamustine. Clin Case Rep 2016;4(1):23-5 doi: 10.1002/ccr3.436[published Online First: Epub Date]|.
137. Usami M, Murase K, Takada K, et al. [Neurolymphomatosis of the sciatic nerve diagnosed by FDG-PET/CT]. Rinsho Ketsueki 2016;57(1):52-5 doi: 10.11406/rinketsu.57.52[published Online First: Epub Date]|.
138. Yu Y, Ren M, Qi X. Pathologically proven peripheral neurolymphomatosis. Neurol India 2016;64(4):805-7 doi: 10.4103/0028-3886.185354[published Online First: Epub Date]|.
139. Yu YX, Liu JY, Yao S. [Diffuse large B-cell lymphoma presenting with neurolymphomatosis: a case report]. Zhonghua Nei Ke Za Zhi 2016;55(9):722-3 doi: 10.3760/cma.j.issn.0578-1426.2016.09.014[published Online First: Epub Date]|.
140. Briani C, Visentin A, Cavallaro T, et al. Primary neurolymphomatosis as clinical onset of chronic lymphocytic leukemia. Ann Hematol 2017;96(1):159-61 doi: 10.1007/s00277-016-2852-2[published Online First: Epub Date]|.
141. Bund C, Heimburger C, Trensz P, Fohrer C, Kremer S, Namer IJ. FDG PET to Diagnose Neurolymphomatosis in a Case of Triple-Hit B-Cell Lymphoma. Clin Nucl Med 2017;42(6):458-60 doi: 10.1097/RLU.0000000000001608[published Online First: Epub Date]|.
142. Byun JM, Kim KH, Kim M, et al. Diagnosis of secondary peripheral neurolymphomatosis: a multi-center experience. Leuk Lymphoma 2017;58(11):2624-32 doi: 10.1080/10428194.2017.1312376[published Online First: Epub Date]|.
143. Chang GY. Evolution of Neurolymphomatosis to Lymphomatosis Cerebri. J Clin Neurol 2017;13(2):203-04 doi: 10.3988/jcn.2017.13.2.203[published Online First: Epub Date]|.
144. de Vaugelade C, de Clermont-Gallerande H, Meyer M, et al. FDG PET/CT in Pediatric Neurolymphomatosis. Clin Nucl Med 2017;42(5):e255-e57 doi: 10.1097/RLU.0000000000001624[published Online First: Epub Date]|.
145. Foo TL, Yak R, Puhaindran ME. Peripheral Nerve Lymphomatosis. J Hand Surg Asian Pac Vol 2017;22(1):104-07 doi: 10.1142/S0218810417720042[published Online First: Epub Date]|.
146. Gorospe L, Gallego-Rivera JI, Rioja-Martin ME, Chinea-Rodriguez A. Bilateral sciatic nerve neurolymphomatosis: PET/CT findings. Ann Hematol 2017;96(6):1059-60 doi: 10.1007/s00277-017-2988-8[published Online First: Epub Date]|.

147. Groth CL, Nevel KS, Gwathmey KG, Bafakih F, Jones DE. Splenic marginal zone lymphoma: An indolent malignancy leading to the development of neurolymphomatosis. Muscle Nerve 2017;55(3):440-44 doi: 10.1002/mus.25404[published Online First: Epub Date]|.
148. Kaulen LD, Foss FM, Fulbright RK, Huttner A, Baehring JM. Neurolymphomatosis of the thoracic sympathetic chain. Neurology 2017;89(18):1926-27 doi: 10.1212/WNL.0000000000004600[published Online First: Epub Date]|.
149. Khader A, Vineetha M, George M, Manakkad SP, Balakrishnan S, Rajan U. Neurolymphomatosis in Primary Cutaneous CD4+ Pleomorphic Small/Medium-sized T-cell Lymphoma Mimicking Hansen's Disease. Indian J Dermatol 2017;62(3):315-17 doi: 10.4103/ijd.IJD_553_16[published Online First: Epub Date]|.
150. Khandelwal S, Saxena S, Hansalia DJ. Neurolymphomatosis: A Surreal Presentation of Lymphoma. Indian J Med Paediatr Oncol 2017;38(3):287-90 doi: 10.4103/ijmpo.ijmpo_151_16[published Online First: Epub Date]|.
151. Laumonerie P, Capek S, Amrami KK, Dyck PJ, Spinner RJ. Targeted fascicular biopsy of the brachial plexus: rationale and operative technique. Neurosurg Focus 2017;42(3):E9 doi: 10.3171/2017.1.FOCUS16404[published Online First: Epub Date]|.
152. Le Guennec L, Maisonobe T, Choquet S, et al. Neurolymphomatosis as a relapse of primary cerebral nervous system lymphoma. Leuk Lymphoma 2017;58(3):729-31 doi: 10.1080/10428194.2016.1211277[published Online First: Epub Date]|.
153. Noda Y, Sekiguchi K, Tokuoka H, et al. Ultrasonographic findings of proximal median neuropathy: A case series of suspected distal neuralgic amyotrophy. J Neurol Sci 2017;377:1-5 doi: 10.1016/j.jns.2017.03.037[published Online First: Epub Date]|.
154. Ono Y, Kazuma Y, Ochi Y, Matsuoka R, Imai Y, Ishikawa T. Two Cases of Neurolymphomatosis with Fatal Bilateral Vocal Cord Paralysis that were Diagnosed with (18)F-fluorodeoxyglucose Positron Emission Tomography (FDG PET)/CT. Intern Med 2017;56(10):1193-98 doi: 10.2169/internalmedicine.56.6998[published Online First: Epub Date]|.
155. Tanimizu M, Kobayashi K. Neurolymphomatosis Appeared Following Primary Central Nervous System Lymphoma. Intern Med 2017;56(4):463-64 doi: 10.2169/internalmedicine.56.7238[published Online First: Epub Date]|.
156. Trevisan AC, Ribeiro FB, Itikawa EN, et al. 18F-FDG PET/CT/MRI Fusion Images Showing Cranial and Peripheral Nerve Involvement in Neurolymphomatosis. Indian J Nucl Med 2017;32(1):77-78 doi: 10.4103/0972-3919.198502[published Online First: Epub Date]|.
157. Yi FF, Luo SS, Zhu WH, Zhao CB. Neurolymphomatosis Caused by Nasal-type Extranodal Natural Killer/T-cell Lymphoma. Chin Med J (Engl) 2017;130(5):625-26 doi: 10.4103/0366-6999.200547[published Online First: Epub Date]|.
158. Asanome A, Kano K, Takahashi K, Saito T, Sawada J, Katayama T. [A case of neurolymphomatosis that was diagnosed by acoustic nerve biopsy]. Rinsho Shinkeigaku 2018;58(2):93-99 doi: 10.5692/clinicalneurol.cn-001080[published Online First: Epub Date]|.
159. Awis Qarni F, Tai E, Wh WH, Husin A. Homonymous Hemianopia: A Rare Presentation of Secondary Central Nervous System Neurolymphomatosis. Cureus 2018;10(5):e2708 doi: 10.7759/cureus.2708[published Online First: Epub Date]|.
160. Bjornard KL, Leventaki V, Nichols KE, Sandlund JT, Prockop S, Ehrhardt MJ. Two-year-old female with EBV-positive diffuse large B-cell lymphoma and subsequent CNS involvement with

neurolymphomatosis. Pediatr Blood Cancer 2018;65(12):e27415 doi: 10.1002/pbc.27415[published Online First: Epub Date]|.

161. Bourque PR, Warman Chardon J, Bryanton M, Toupin M, Burns BF, Torres C. Neurolymphomatosis of the Brachial Plexus and its Branches: Case Series and Literature Review. Can J Neurol Sci 2018;45(2):137-43 doi: 10.1017/cjn.2017.282[published Online First: Epub Date]|.

162. Brand C, Pala A, Scheuerle A, et al. [Neurolymphomatosis : Two case reports]. Nervenarzt 2018;89(6):701-04 doi: 10.1007/s00115-017-0460-6[published Online First: Epub Date]|.

163. Daher A, Kamiya-Matsuoka C, Woodman K. Patient With 2 Hematologic Malignancies Presenting as Neurolymphomatosis. J Clin Neuromuscul Dis 2018;19(3):124-30 doi: 10.1097/CND.0000000000000183[published Online First: Epub Date]|.

164. Davidson T, Kedmi M, Avigdor A, et al. FDG PET-CT evaluation in neurolymphomatosis: imaging characteristics and clinical outcomes. Leuk Lymphoma 2018;59(2):348-56 doi: 10.1080/10428194.2017.1352096[published Online First: Epub Date]|.

165. Duchesne M, Roussellet O, Maisonobe T, et al. Pathology of Nerve Biopsy and Diagnostic Yield of PCR-Based Clonality Testing in Neurolymphomatosis. J Neuropathol Exp Neurol 2018;77(9):769-81 doi: 10.1093/jnen/nly055[published Online First: Epub Date]|.

166. Haydaroglu Sahin H, Mete A, Pehlivan M. Neurolymphomatosis in non-Hodgkin lymphoma with cranial multineuritis: A case report. Medicine (Baltimore) 2018;97(15):e0303 doi: 10.1097/MD.0000000000010303[published Online First: Epub Date]|.

167. Jimenez Zapata HD, Rojas Medina LM, Carrasco Moro R, Martinez Rodrigo A, Garcia-Cosio Piqueras M. Cauda equina syndrome secondary to neurolymphomatosis: Case report and literature review. Neurocirugia (Astur : Engl Ed) 2018;29(3):138-42 doi: 10.1016/j.neucir.2017.07.001[published Online First: Epub Date]|.

168. Kobayashi M, Sakai Y, Kariya Y, et al. First pathological report of a de novo CD5-positive diffuse large B-cell lymphoma patient presenting with Guillain-Barre syndrome-like neuropathy due to neurolymphomatosis. Neuropathology 2018 doi: 10.1111/neup.12470[published Online First: Epub Date]|.

169. Konishi H, Taguchi Y, Yamamoto M, Nukui T, Dougu N, Nakatsuji Y. [A case of neurolymphomatosis presented as cauda equine syndrome accompanied with M-proteinemia]. Rinsho Shinkeigaku 2018;58(4):223-28 doi: 10.5692/clinicalneurol.cn-001079[published Online First: Epub Date]|.

170. Liu KC, Hennessey MA, McCall CM, Proia AD. Ocular involvement in neurolymphomatosis. Am J Ophthalmol Case Rep 2018;10:148-51 doi: 10.1016/j.ajoc.2018.02.023[published Online First: Epub Date]|.

171. Mahmoud RA, Abrams CK. Acute demyelinating neuropathy in a patient with neurolymphomatosis. BMJ Case Rep 2018;2018 doi: 10.1136/bcr-2017-222814[published Online First: Epub Date]|.

172. Mustafa R, Klein CJ, Martinez-Thompson J, et al. Recurrent Brachial Neuritis Attacks in Presentation of B-Cell Lymphoma. Mayo Clin Proc Innov Qual Outcomes 2018;2(4):382-86 doi: 10.1016/j.mayocpiqo.2018.10.002[published Online First: Epub Date]|.

173. Park HJ, Shin HY, Kim SH, et al. Partial Conduction Block as an Early Nerve Conduction Finding in Neurolymphomatosis. J Clin Neurol 2018;14(1):73-80 doi: 10.3988/jcn.2018.14.1.73[published Online First: Epub Date]|.

174. Phang I, Craig-Mcquaide A, Kinch K, Taylor W. Burkitt's neurolymphomatosis of the trigeminal nerve. BMJ Case Rep 2018;2018 doi: 10.1136/bcr-2018-225958[published Online First: Epub Date]|.
175. Roy SG, Parida GK, Tripathy S, Das CJ, Kumar R. Active Leprosy Neuritis Detected on FDG PET/CT. Clin Nucl Med 2018;43(2):132-33 doi: 10.1097/RLU.0000000000001946[published Online First: Epub Date]|.
176. Sasaki R, Ohta Y, Yamada Y, et al. Neurolymphomatosis in the Cauda Equina Diagnosed by an Open Biopsy. Intern Med 2018;57(23):3463-65 doi: 10.2169/internalmedicine.1049-18[published Online First: Epub Date]|.
177. Schuster NM, Volney SJ, Kamdar MM. Sciatic Lymphoma Mimicking Lumbar Radiculopathy. Pain Med 2018;19(10):2091-92 doi: 10.1093/pm/pny153[published Online First: Epub Date]|.
178. Uematsu N, Sumi M, Kaiume H, et al. [Neurolymphomatosis due to enteropathy-associated T-cell lymphoma clinically diagnosed by FDG-PET/CT and subsequently confirmed by autopsy]. Rinsho Ketsueki 2018;59(1):69-74 doi: 10.11406/rinketsu.59.69[published Online First: Epub Date]|.
179. Bourque PR, Sampaio ML, Warman-Chardon J, Samaan S, Torres C. Neurolymphomatosis of the lumbosacral plexus and its branches: case series and literature review. BMC Cancer 2019;19(1):1149 doi: 10.1186/s12885-019-6365-y[published Online First: Epub Date]|.
180. Chague P, Phan CM, Lapusan S, Zhang-Yin J, Cottereau AS. (18)F-FDG PET/CT imaging findings of extensive neurolymphomatosis as a relapse of diffuse large B cell lymphoma. Diagn Interv Imaging 2019;100(9):527-28 doi: 10.1016/j.diii.2019.02.006[published Online First: Epub Date]|.
181. DeBoer SR, Lesche S, Rodriguez FJ, Ostrow LW. Teaching NeuroImages: Neurolymphomatosis. Neurology 2019;93(12):e1229-e30 doi: 10.1212/WNL.0000000000008131[published Online First: Epub Date]|.
182. DeVries AH, Howe BM, Spinner RJ, Broski SM. B-cell peripheral neurolymphomatosis: MRI and (18)F-FDG PET/CT imaging characteristics. Skeletal Radiol 2019;48(7):1043-50 doi: 10.1007/s00256-019-3145-3[published Online First: Epub Date]|.
183. Ganeshalingam R, Roach P, Schembri GP. Diffuse Large B-Cell Lymphoma Recurring as Neurolymphomatosis on FDG PET/CT. Clin Nucl Med 2019;44(2):145-47 doi: 10.1097/RLU.0000000000002388[published Online First: Epub Date]|.
184. Iacobellis F, Di Serafino M, Blasio R, Barbuto L, Pezzullo F, Romano L. Secondary Neurolymphomatosis of the Radial Nerve: A Diagnostic Challenge. Am J Case Rep 2019;20:1652-58 doi: 10.12659/AJCR.916961[published Online First: Epub Date]|.
185. Kobayashi H, Abe Y, Miura D, et al. Limited efficacy of high-dose methotrexate in patients with neurolymphomatosis. Int J Hematol 2019;109(3):286-91 doi: 10.1007/s12185-018-02586-7[published Online First: Epub Date]|.
186. Li H, Luo SS, Zhao CB. Neurolymphomatosis caused by diffuse large B-cell lymphoma presenting as isolated brachial plexopathy. Chin Med J (Engl) 2019;132(22):2762-64 doi: 10.1097/CM9.0000000000000514[published Online First: Epub Date]|.
187. Li V, Jaunmuktane Z, Cwynarski K, Carr A. Diagnostic delay in a case of T-cell neurolymphomatosis. BMJ Case Rep 2019;12(12) doi: 10.1136/bcr-2019-232538[published Online First: Epub Date]|.

188. Matsue K, Abe Y, Narita K, et al. Diagnosis of intravascular large B cell lymphoma: novel insights into clinicopathological features from 42 patients at a single institution over 20 years. Br J Haematol 2019;187(3):328-36 doi: 10.1111/bjh.16081[published Online First: Epub Date]|.
189. Mori Y, Yamamoto K, Ohno A, Fukunaga M, Nishikawa A. Primary Central Nervous System Lymphoma with Peripheral Nerve Involvement: Case Report. Cureus 2019;11(9):e5675 doi: 10.7759/cureus.5675[published Online First: Epub Date]|.
190. Narita K, Kobayashi H, Kitadate A, et al. Neurolymphomatosis of the sciatic and tibial nerves as an initial presentation of lung diffuse large B cell lymphoma detected by positron emission tomography/computed tomography. Int J Hematol 2019;110(4):385-86 doi: 10.1007/s12185-019-02705-y[published Online First: Epub Date]|.
191. Padilha IG, Fischer T, Pacheco FT, Da Rocha AJ. Brachial and sacral plexus neurolymphomatosis - unusual regions for disease relapses. Arq Neuropsiquiatr 2019;77(11):832-33 doi: 10.1590/0004-282X20190104[published Online First: Epub Date]|.
192. Puffer RC, Dyck PJB, Paul P, Broski SM, Amrami KK, Spinner RJ. Putative mechanisms for spread and transformation of cutaneous T-cell lymphoma to neurolymphomatosis. Muscle Nerve 2019;60(5):E30-E33 doi: 10.1002/mus.26670[published Online First: Epub Date]|.
193. Rai W, Olcese V, Elsheikh B, Stino AM. Horner's Syndrome as Initial Manifestation of Possible Brachial Plexopathy Neurolymphomatosis. Front Neurol 2019;10:4 doi: 10.3389/fneur.2019.00004[published Online First: Epub Date]|.
194. Sato H, Hiroshima S, Anei R, Kamada K. Primary neurolymphomatosis of the trigeminal nerve. Br J Neurosurg 2019:1-4 doi: 10.1080/02688697.2019.1568391[published Online First: Epub Date]|.
195. Sosa-Albacete F, Pappolla A, Hem S, et al. [Primary neurolymphomatosis in the cauda equina as the initial symptom of human immunodeficiency virus]. Rev Neurol 2019;69(7):301-02 doi: 10.33588/rn.6907.2019244[published Online First: Epub Date]|.
196. Tai R, Maingard J, Nambiar M, Lim K. High-grade B-cell lymphoma relapse presenting as neurolymphomatosis of the median nerve. BMJ Case Rep 2019;12(3) doi: 10.1136/bcr-2018-228742[published Online First: Epub Date]|.
197. Yaseen MT, Aziz PAA, Siddique K, Tariq TA. Neurolymphomatosis of the Sciatic and Tibial Nerves. J Coll Physicians Surg Pak 2019;29(12):S86-S88 doi: 10.29271/jcpsp.2019.12.S86[published Online First: Epub Date]|.
198. Campagnolo M, Cacciavillani M, Cavallaro T, et al. Neurolymphomatosis, a rare manifestation of peripheral nerve involvement in lymphomas: Suggestive features and diagnostic challenges. J Peripher Nerv Syst 2020;25(3):312-15 doi: 10.1111/jns.12401[published Online First: Epub Date]|.
199. Facchinelli D, Ciliberti E, Stussi G, Ceriani L, Zucca E. Sciatic pain by neurolymphomatosis as initial presentation of disseminated diffuse large B cell lymphoma involving the testis and the CNS. Hematol Oncol 2020;38(2):197-200 doi: 10.1002/hon.2698[published Online First: Epub Date]|.
200. Fatima N, Zaman MU, Zaman A, Zaman SU. Neurolymphomatosis - Rare presentation in non-Hodgkin's lymphoma: The role of (18)F-fluorodeoxyglucose positron-emission tomography and computerized tomography imaging. World J Nucl Med 2020;19(2):159-61 doi: 10.4103/wjnm.WJNM_22_19[published Online First: Epub Date]|.

201. Fitzgerald L, Stephens DM. Burkitt Lymphoma Presenting as Cranial Multineuritis Secondary to Primary Neurolymphomatosis: A Diagnostic Challenge. Clin Lymphoma Myeloma Leuk 2020;20(4):e201-e04 doi: 10.1016/j.clml.2020.01.012[published Online First: Epub Date]|.
202. Keddie S, Nagendran A, Cox T, et al. Peripheral nerve neurolymphomatosis: Clinical features, treatment, and outcomes. Muscle Nerve 2020;62(5):617-25 doi: 10.1002/mus.27045[published Online First: Epub Date]|.
203. Lorance DK, Allison JB, Gardner JA, Waheed W. Protean Manifestations and Diagnostic Challenges Including Discordance Between Electrodiagnostic-Radiologic Studies in Neurolymphomatosis. Neurologist 2020;25(1):4-9 doi: 10.1097/NRL.0000000000000245[published Online First: Epub Date]|.
204. Murthy NK, Amrami KK, Spinner RJ. Circumdural extension of perineural spread leading to bilateral disease in neurolymphomatosis. Acta Neurochir (Wien) 2020;162(12):3197-200 doi: 10.1007/s00701-020-04417-3[published Online First: Epub Date]|.
205. Murthy NK, Hebert-Blouin MN, Capek S, Prasad NK, Amrami KK, Spinner RJ. Understanding the Pathognomonic Tumefactive Appearance of Neurolymphomatosis: A Unifying Theory of Neurolymphoma. World Neurosurg 2020;141:e490-e97 doi: 10.1016/j.wneu.2020.05.228[published Online First: Epub Date]|.
206. Murthy NK, Sharma M, Spinner RJ. Primary peripheral nerve tumors associated with nerve-territory herpes zoster. Acta Neurochir (Wien) 2020;162(5):1147-51 doi: 10.1007/s00701-020-04292-y[published Online First: Epub Date]|.
207. Nepal P, Batchala PP, Rehm PK, Fadul CE. Diffuse large B-cell lymphoma relapse presenting as extensive neurolymphomatosis. Neuroradiol J 2020;33(3):230-35 doi: 10.1177/1971400920924799[published Online First: Epub Date]|.
208. Nishikawara M, Kawakami T, Sakai H, et al. Magnetic Resonance Imaging-negative, Rituximab-resistant Neurolymphomatosis as a Paradoxical Presentation of Relapsed Primary Adrenal Lymphoma. Intern Med 2020;59(11):1437-43 doi: 10.2169/internalmedicine.4085-19[published Online First: Epub Date]|.
209. Pan Q, Luo Y. Recurrence of nasal type NK/T cell lymphoma presenting as neurolymphomatosis on 18F-FDG PET/CT: A case report and literature review. Medicine (Baltimore) 2020;99(1):e18640 doi: 10.1097/MD.0000000000018640[published Online First: Epub Date]|.
210. Seegobin K, Alhaj Moustafa M, Fischer D, et al. Systemic ALK-positive anaplastic large cell lymphoma with bilateral optic neurolymphomatosis resulting in permanent blindness. Clin Case Rep 2020;8(12):2629-33 doi: 10.1002/ccr3.3231[published Online First: Epub Date]|.
211. Shima T, Tsujino A. [Neurolymphomatosis Mimicking Inflammatory Neuropathy with Slight FDG Uptake on PET/CT and Distinct MRI Abnormalities in the Brachial Plexus: A Case Report]. Brain Nerve 2020;72(9):987-92 doi: 10.11477/mf.1416201632[published Online First: Epub Date]|.
212. Singh SS, Mittal BR, Kumar R, Singh H, Balaini N, Goyal M. Primary Central Nervous System Lymphoma With Diffuse Neurolymphomatosis Involving Multiple Cranial and Spinal Nerve Roots. Clin Nucl Med 2020;45(6):e285-e87 doi: 10.1097/RLU.0000000000003018[published Online First: Epub Date]|.
213. Switlyk MD, Skeie AT, Lund-Iversen M, Ostenstad B. Magnetic resonance imaging and (18)F-fluorodeoxyglucose positron emission tomography/computed tomography findings in

neurolymphomatosis: an uncommon presentation of diffuse large B cell lymphoma. Ann Hematol 2020;99(1):203-05 doi: 10.1007/s00277-019-03850-4[published Online First: Epub Date]|.
214. Yadav D, Angamuthu M, Subudhi TK, Das CJ, Kumar R. PET/CT in Evaluation of Low Backache Due to Sacral Nerve Root Involvement: Inflammatory and Malignant Involvement. Clin Nucl Med 2020;45(6):e299-e300 doi: 10.1097/RLU.0000000000003021[published Online First: Epub Date]|.
215. Alazawi S, Elomri H, Taha R, et al. Neurolymphomatosis of the median nerve, optic nerve, L4 spinal nerve root and cauda equina in patients with B-cell malignancies: a case series. J Med Case Rep 2021;15(1):133 doi: 10.1186/s13256-021-02714-8[published Online First: Epub Date]|.
216. Campagnolo M, Cacciavillani M, Briani C. Heterogeneous clinical and imaging findings and long-term prognosis in patients with neurolymphomatosis. Muscle Nerve 2021;63(3):E24-E26 doi: 10.1002/mus.27154[published Online First: Epub Date]|.
217. Fritzhand SJ, Esmaeli B, Sun J, Debnam JM. Primary disease sites and patterns of spread in cases of neurolymphomatosis in the orbit associated with lymphoma. Cancer Imaging 2021;21(1):39 doi: 10.1186/s40644-021-00409-3[published Online First: Epub Date]|.
218. Hamaguchi M, Kokubun N, Matsuda H, et al. A case report of secondary neurolymphomatosis showing selective nerve infiltration and massive lumbar plexus enlargement. BMC Neurol 2021;21(1):296 doi: 10.1186/s12883-021-02330-5[published Online First: Epub Date]|.
219. Jeong J, Kim SW, Sung DH. Neurolymphomatosis: a single-center experience of neuromuscular manifestations, treatments, and outcomes. J Neurol 2021;268(3):851-59 doi: 10.1007/s00415-020-10202-0[published Online First: Epub Date]|.
220. Khurana A, Dalland JC, Young JR, Inwards DJ, Paludo J. Brexucabtagene autoleucel therapy induces complete remission in a primary refractory blastoid mantle cell lymphoma with neurolymphomatosis. Am J Hematol 2021;96(8):E298-E301 doi: 10.1002/ajh.26233[published Online First: Epub Date]|.
221. Khurana A, Novo M, Nowakowski GS, et al. Clinical manifestations of, diagnostic approach to, and treatment of neurolymphomatosis in the rituximab era. Blood Adv 2021;5(5):1379-87 doi: 10.1182/bloodadvances.2020003666[published Online First: Epub Date]|.
222. Kim KT, Kim SI, Do YR, Jung HR, Cho JH. Sciatic nerve neurolymphomatosis as the initial presentation of primary diffuse large B-cell lymphoma: a rare cause of leg weakness. Yeungnam Univ J Med 2021;38(3):258-63 doi: 10.12701/yujm.2021.00983[published Online First: Epub Date]|.
223. Kuhlman JJ, Alhaj Moustafa M, Gupta V, Jiang L, Tun HW. Primary Cauda Equina Lymphoma Treated with CNS-Centric Approach: A Case Report and Literature Review. J Blood Med 2021;12:645-52 doi: 10.2147/JBM.S325264[published Online First: Epub Date]|.
224. Lee YC, Huang GS, Chang WC, Hsu YC. Fried egg sign: A typical ultrasonography feature of neurolymphomatosis. J Clin Ultrasound 2021;49(8):878-80 doi: 10.1002/jcu.23029[published Online First: Epub Date]|.
225. Makranz C, Arkadir D, Nachmias B, et al. Neurological misdiagnoses of lymphoma. Neurol Sci 2021;42(5):1933-40 doi: 10.1007/s10072-020-04724-8[published Online First: Epub Date]|.
226. Negre Buso M, Balliu Collgros E, Rubio Rodriguez A, Pelaez Hernandez I, Mestre Fusco A, Roncero Vidal JM. Utility of the (18)F-FDG PET/CT in the diagnosis of the

neurolymphomatosis: A case report. Rev Esp Med Nucl Imagen Mol (Engl Ed) 2021;40(5):328-31 doi: 10.1016/j.remnie.2020.09.017[published Online First: Epub Date]|.
227. Nishida K, Takenaka Y, Yamasaki H, Futamura N. Garcin Syndrome in a Patient With Neurolymphomatosis. Neurology 2021 doi: 10.1212/WNL.0000000000012485[published Online First: Epub Date]|.
228. Rastogi P, Singh H, Gupta P, Bal A, Dutta U, Gupta N. Extra-hepatic biliary obstruction due to diffuse large B-cell lymphoma: an autopsy case report with unattended yet significant pathologies. Clin J Gastroenterol 2021;14(1):269-74 doi: 10.1007/s12328-020-01252-y[published Online First: Epub Date]|.
229. Raut TP, Bhatt M, Hastak M, et al. Neurolymphomatosis as a Presenting Feature of Primary Testicular Lymphoma. Ann Indian Acad Neurol 2021;24(2):269-72 doi: 10.4103/aian.AIAN_304_20[published Online First: Epub Date]|.
230. Sheng S, Sharma R, Samant R, Yuan J, Nalleballe K, Kapoor N. Isolated primary neurolymphomatosis with cranial multineuritis: a case presentation. Neurol Sci 2021;42(3):1223-26 doi: 10.1007/s10072-020-04806-7[published Online First: Epub Date]|.
231. Barahona D, Adlerstein I, Donoso J, Mercado F. Sciatic and median nerve neurolymphomatosis as initial presentation of B-cell lymphoma. Radiologia (Engl Ed) 2022;64(3):266-69 doi: 10.1016/j.rxeng.2020.10.003[published Online First: Epub Date]|.

Supplemental Table 2: Lists of peripheral nerve involvement.

Cranial Nerves(226)	
Unspecified cranial nerves	129
Olfactory	1
Optic Nerve	4
Oculomotor nerve	12
Trochlear nerve	1
Trigeminal nerve	32
Mandibular nerve	1
Abducens nerve	5
Facial nerves	16
Mental nerve	2
Temproal nerve	1
Vestibulocochlear (acoustic)	7
Vagus nerve	5
Hypoglossal nerve	1
Glossopharyngeal	3
Infratrochlear nerve	1
Pharyngeal/vagal nerve	1
Ciliary nerve	1
Internal auditory canal nerve	1
7th and 8th nerve complex	1
7th and 8th nerve complex	1
Neural Plexus(290)	
Unspecified Peripheral nerves	27
Brachial plexus(139)	
Brachial plexus	133
Axilary nerve	5
Suprascapular nerve	1
lumbosacral plexus nerves(151)	
Lumbosacral plexus nerves	71
Lumbar plexus	17
Obturator nerve	1
Piriformis nerve	1
Cauda Equina	59
Iliac nerves	1
Gluteal nerve	1
Spinal Nerves(241)	
Unspecified Spinal nerves	45
Lumbar and sacral nerve roots(92)	
Lumbar nerves (L)	37
Sacral (S)	50
S1	1
S1-S3	2
S2	1
Coccygeal	1
Lumbar & sacral nerve roots(61)	
Cervical spinal nerves (C)	54
C4/5	1
C5/6	1
C8	5
Thoracic nerve roots(43)	
Thoracic nerves	38
T8-9	1
T12-L1	0
T12-L5	1
Intercostal	3
Peripheral Nerves(338)	
Distal upper extremities nerves(44)	
Median Nerve	27
Anterior interosseous nerve	1
Ulnar nerve	12
Radial nerve	4
Posterior cutaneous nerve of forearm	0
Distal lower extremity nerves(294)	
Saphenous nerve	3
Femoral nerve	30
Sciatic	120
Peroneal nerve	17
Fibular nerves	1
Popliteal nerve	2
Sural nerve	15
Tibial (shin)	25
Plantar nerves	0
Unspecified neural plexus	81
Other nerves(8)	
Other nerves	8

Supplemental Table 3: Lists of lymphoma histologies. ALCL, anaplastic large cell lymphoma; CLL/SLL, chronic lymphocytic leukemia/small lymphocytic lymphoma; IVL, intravascular lymphomatosis; MALT, mucosa-assisted lymphoid tissue; NK, natural killer.

B-Cell lymphomas(689)	
Unspecified B-cell	18
Diffuse large B-cell	360
Follicular	51
CLL/SLL	10
Mantle cell	51
Marginal zone	50
Burkitt	13
Lymphoplasmacytic	53
MALT	5
High-grade B-cell	10
Low-grade B-cell	46
Intermediate & triple-hit B-cell	3
B acute lymphoblastic leukemia	1
IVL	18
Plasma cell leukemia	1
**T-cell lymphomas(91)**	
T lymphoblastic lymphoma/leukemia	8
Peripheral T-cell	46
Cutaneous T-cell	4
Adult T-cell leukemia/lymphoma	2
Extranodal NK/T-cell* (lymphoma, nasal type)	8
ALCL	2
Unspecified T-cell	14
Enteropathy-associated T-cell	1
T-cell acute lymphocytic leukemia	1
**Other(5)**	
Acute nonlymphocytic leukemia	2
Acute myelogenous leukemia	2
Histiocytic lymphoma	1

Supplemental Table 4: Decade analysis of (A) OS, (B) treatment 1 to progression, (C) symptom onset to diagnosis, and (D) symptom onset to death periods.

**Decade Analysis**						
**Overall Survival**						
**Decades**	**N**	**Median (months)**	**Mean (months)**	**Standard deviation**	**Lower bound**	**Upper bound**
≤1990	8	0	0.25	0.707106781	0	1.664213562
1991-2000	14	0.5	7.535714286	22.11374815	0	51.76321058
2001-2010	93	10	10.22768421	12.6038322	0	35.43534861
2011-2020	248	8	14.76141129	19.952725	0	54.66686129
2021-2022	50	26.2	73.288	58.07474649	0	189.437493
**Time from Treatment 1 to Progression**						
**Decades**	**N**	**Median (months)**	**Mean (months)**	**Standard deviation**	**Lower bound**	**Upper bound**
≤1990	0	N/A	N/A	N/A	N/A	N/A
1991-2000	1	2	2	N/A	N/A	N/A
2001-2010	4	2	4.1875	5.304773793	0	14.79704759
2011-2020	49	6.4	9.305918367	11.57894244	0	32.46380324
2021-2022	40	36	28.61	11.04609921	6.517801586	50.70219841
**Time from Symptom Onset to Diagnosis**						
**Decades**	**N**	**Median (months)**	**Mean (months)**	**Standard deviation**	**Lower bound**	**Upper bound**
≤1990	1	6	6	N/A	N/A	N/A
1991-2000	N/A	N/A	N/A	N/A	N/A	N/A
2001-2010	20	2	9.2	17.81143926	0	44.82287851
2011-2020	81	3	7.821851852	14.76708224	0	37.35601632
2021-2022	7	4	18.78571429	34.62881404	0	88.04334237
**Time from Symptom Onset to Death**						
**Decades**	**N**	**Median (months)**	**Mean (months)**	**Standard deviation**	**Lower bound**	**Upper bound**
≤1990	8	0	1	2.138089935	0	5.276179871
1991-2000	14	0.5	7.535714286	22.11374815	0	51.76321058
2001-2010	95	10	12.16136842	19.06360019	0	50.28856881
2011-2020	266	8.75	16.1443609	21.13840025	0	58.4211614
2021-2022	52	26.2	72.99807692	57.50248585	0	188.0030486
NA= Not Available						

Supplemental Table 5: Analysis of various treatments on (A) OS, (B) treatment 1 to progression, (C) symptom onset to diagnosis, and (D) symptom onset to death periods.

5A

Overall Survival						
Types of treatment	N	Median (months)	Mean (months)	tandard deviatic	Lower bound	Upper bound
Rituximab & R-CHOP	125	16.25	32.472	44.37503757	0	121.2220751
HD-MTX	81	18.5	37.22098765	47.24352697	0	131.7080416
Radiation	79	6.885	11.70594937	17.20808429	0	46.12211795
HD-MTX + rituximab	40	19	19.5	11.97914855	0	43.4582971
Transplant	32	25.4	58.5915625	55.94084605	0	170.4732546
HD-MTX + transplant	12	25.4	50.61666667	52.40520991	0	155.4270865
Rituximab + transplant	11	23.65	60.87272727	60.21908235	0	181.310892
Corticosteroids	6	12	12.66666667	6.047431568	0.57180353	24.7615298
HD-MTX + rituximab + transplant	5	138	88.9	69.22746324	0	227.3549265
**Kruskal-Wallis chi-squared = 63.493**	**df = 8**	**p value = 9.578e-11**				

Pair	Z score	Bonferroni adjusted p value
Radiation vs. Transplant	-6.093421218	1.98941E-08
HD-MTX vs. Radiation	5.419037395	1.07857E-06
Radiation vs. Rituximab & R-CHOP	-4.584609307	8.18705E-05
HD-MTX + transplant vs. Radiation	4.101924519	0.00073751
HD-MTX + rituximab vs. Radiation	4.043568706	0.000947587
Radiation vs. Rituximab + transplant	-3.951242233	0.001399438
HD-MTX + rituximab + transplant vs. Radiation	3.413612016	0.0115394
Rituximab & R-CHOP vs. Transplant	-3.118820434	0.03268379
HD-MTX + rituximab vs. Transplant	-2.075083322	0.6836186
HD-MTX + transplant vs. Rituximab & R-CHOP	2.024837769	0.7719125
HD-MTX vs. Transplant	-2.01125314	0.7973771
HD-MTX + rituximab + transplant vs. Rituximab & R-CHOP	2.006793363	0.8058901
Corticosteroids vs. Transplant	-1.999593993	0.8197942
Corticosteroids vs. HD-MTX + rituximab + transplant	-1.960143547	0.8996223
Rituximab & R-CHOP vs. Rituximab + transplant	-1.947990314	0.9254902
Corticosteroids vs. HD-MTX	-1.109989084	1
Corticosteroids vs. HD-MTX + rituximab	-0.907786497	1
HD-MTX vs. HD-MTX + rituximab	0.373650378	1
HD-MTX vs. HD-MTX + rituximab + transplant	-1.556588261	1
HD-MTX + rituximab vs. HD-MTX + rituximab + transplant	-1.664410425	1
Corticosteroids vs. HD-MTX + transplant	-1.767243395	1
HD-MTX vs. HD-MTX + transplant	-1.338376601	1
HD-MTX + rituximab vs. HD-MTX + transplant	-1.477166519	1
HD-MTX + rituximab + transplant vs. HD-MTX + transplant	0.569809304	1
Corticosteroids vs. Radiation	0.914490685	1
Corticosteroids vs. Rituximab & R-CHOP	-0.650076212	1
HD-MTX vs. Rituximab & R-CHOP	1.387758267	1
HD-MTX + rituximab vs. Rituximab & R-CHOP	0.692172641	1
Corticosteroids vs. Rituximab + transplant	-1.74244955	1
HD-MTX vs. Rituximab + transplant	-1.290537126	1
HD-MTX + rituximab vs. Rituximab + transplant	-1.430148019	1
HD-MTX + rituximab + transplant vs. Rituximab + transplant	0.561033981	1
HD-MTX + transplant vs. Rituximab + transplant	-0.001688781	1
HD-MTX + rituximab + transplant vs. Transplant	0.61833954	1
HD-MTX + transplant vs. Transplant	-0.017589872	1
Rituximab + transplant vs. Transplant	-0.015018806	1

5B

Time from Treatment 1 to Progression						
**Types of treatment**	**N**	**Median (months)**	**Mean (months)**	**andard deviatio**	**Lower bound**	**Upper bound**
Rituximab & R-CHOP	51	36	23.431	15.01322302	0	53.45744605
HD-MTX	45	36	24.75340909	14.62797387	0	54.00935683
HD-MTX + rituximab	31	36	32.06666667	11.31299567	9.440675332	54.692658
Transplant	19	11	17.25555556	11.0250003	0	39.30555615
Radiation	14	9.4	7.861538462	3.521741993	0.818054475	14.90502245
Rituximab + transplant	7	36	26.5	16.47888259	0	59.45776518
HDMTX + transplant	6	28.4	29.92	14.17072771	1.578544582	58.26145542
Corticosteroids	1	13	N/A	8.485281374	N/A	N/A
HD-MTX + rituximab + transplant	0	N/A	N/A	N/A	N/A	N/A
N/A = Not applicable						
**Kruskal-Wallis chi-squared = 26.8**	**df = 7**	**p value = 0.0003621**				
**Pair**	**Z score**	**Bonferroni adjusted p value**				
HD-MTX + rituximab vs. Radiation	4.9307755	1.14665E-05				
HD-MTX vs. Radiation	3.6202112	0.004121077				
Radiation vs. Rituximab & R-CHOP	-3.3744642	0.01035433				
HD-MTX + rituximab vs. Transplant	3.0069485	0.03694382				
HD-MTX + rituximab vs. Rituximab & R-CHOP	2.5194367	0.1645599				
HDMTX + transplant vs. Radiation	2.4217916	0.2162189				
Radiation vs. Rituximab + transplant	-2.2129991	0.3765679				
HD-MTX vs. HD-MTX + rituximab	-2.0773454	0.5287757				
Radiation vs. Transplant	-2.0204051	0.6067794				
Corticosteroids vs. HD-MTX	-0.4078558	1				
Corticosteroids vs. HD-MTX + rituximab	-0.880889	1				
Corticosteroids vs. HDMTX + transplant	-0.4526959	1				
HD-MTX vs. HDMTX + transplant	-0.17671	1				
HD-MTX + rituximab vs. HDMTX + transplant	0.9103312	1				
Corticosteroids vs. Radiation	0.669256	1				
Corticosteroids vs. Rituximab & R-CHOP	-0.3202304	1				
HD-MTX vs. Rituximab & R-CHOP	0.4395543	1				
HDMTX + transplant vs. Rituximab & R-CHOP	0.38425	1				
Corticosteroids vs. Rituximab + transplant	-0.3102524	1				
HD-MTX vs. Rituximab + transplant	0.1986457	1				
HD-MTX + rituximab vs. Rituximab + transplant	1.3461263	1				
HDMTX + transplant vs. Rituximab + transplant	0.2827251	1				
Rituximab & R-CHOP vs. Rituximab + transplant	-0.0208117	1				
Corticosteroids vs. Transplant	-0.0184066	1				
HD-MTX vs. Transplant	1.4424866	1				
HDMTX + transplant vs. Transplant	1.003822	1				
Rituximab & R-CHOP vs. Transplant	1.1355556	1				
Rituximab + transplant vs. Transplant	0.7074415	1				

5C

Time from Symptom Onset to Diagnosis						
**Types of treatment**	**N**	**Median (months)**	**Mean (months)**	**andard deviatic**	**Lower bound**	**Upper bound**
Rituximab & R-CHOP	35	3	11.02941176	20.3533469	0	51.73610557
Radiation	22	2.566666667	8.434126984	10.9523765	0	30.33887998
HD-MTX	16	3.25	5.5	5.755751599	0	17.0115032
Transplant	8	5.5	18.85714286	30.25768956	0	79.37252199
HD-MTX + rituximab	8	4	5.857142857	4.841946349	0	15.54103555
HD-MTX + transplant	4	8	9.666666667	5.93295879	0	21.53258425
Rituximab + transplant	3	8	52	45.90842334	0	143.8168467
Corticosteroids	1	6	N/A	3.535533906	N/A	N/A
HD-MTX + rituximab + transplant	1	8	N/A	4.949747468	N/A	N/A
N/A = Not applicable						

**Kruskal-Wallis chi-squared = 3.9978**	**df =8**	**p value =0.8573**

**Pair**	**Z score**	**Bonferroni adjusted p value**
Corticosteroids vs. HD-MTX	0.40138776	1
Corticosteroids vs. HD-MTX + rituximab	0.41719656	1
HD-MTX vs. HD-MTX + rituximab	0.06642472	1
Corticosteroids vs. HD-MTX + rituximab + transplant	-0.23780204	1
HD-MTX vs. HD-MTX + rituximab + transplant	-0.72764947	1
HD-MTX + rituximab vs. HD-MTX + rituximab + transplant	-0.73426595	1
Corticosteroids vs. HD-MTX + transplant	-0.25330394	1
HD-MTX vs. HD-MTX + transplant	-1.24673035	1
HD-MTX + rituximab vs. HD-MTX + transplant	-1.18507326	1
HD-MTX + rituximab + transplant vs. HD-MTX + transplant	0.04749449	1
Corticosteroids vs. Radiation	0.44458025	1
HD-MTX vs. Radiation	0.12427102	1
HD-MTX + rituximab vs. Radiation	0.02923084	1
HD-MTX + rituximab + transplant vs. Radiation	0.77349095	1
HD-MTX + transplant vs. Radiation	1.35730763	1
Corticosteroids vs. Rituximab & R-CHOP	0.31015736	1
HD-MTX vs. Rituximab & R-CHOP	-0.328663	1
HD-MTX + rituximab vs. Rituximab & R-CHOP	-0.32649327	1
HD-MTX + rituximab + transplant vs. Rituximab & R-CHOP	0.64175646	1
HD-MTX + transplant vs. Rituximab & R-CHOP	1.13255192	1
Radiation vs. Rituximab & R-CHOP	-0.51461541	1
Corticosteroids vs. Rituximab + transplant	-0.16350699	1
HD-MTX vs. Rituximab + transplant	-0.95770468	1
HD-MTX + rituximab vs. Rituximab + transplant	-0.93249986	1
HD-MTX + rituximab + transplant vs. Rituximab + transplant	0.12773984	1
HD-MTX + transplant vs. Rituximab + transplant	0.12359967	1
Radiation vs. Rituximab + transplant	-1.04535849	1
Rituximab & R-CHOP vs. Rituximab + transplant	-0.83672035	1
Corticosteroids vs. Transplant	0.04171966	1
HD-MTX vs. Transplant	-0.85330212	1
HD-MTX + rituximab vs. Transplant	-0.7965068	1
HD-MTX + rituximab + transplant vs. Transplant	0.35878904	1
HD-MTX + transplant vs. Transplant	0.53472818	1
Radiation vs. Transplant	-0.99384848	1
Rituximab & R-CHOP vs. Transplant	-0.68976544	1
Rituximab + transplant vs. Transplant	0.3442406	1

5D

Time from Symptom Onset to Death

Types of treatment	N	Median (months)	Mean (months)	tandard deviatio	Lower bound	Upper bound
Rituximab & R-CHOP	125	18	34.572	44.22640267	0	123.0248053
HD-MTX	81	20.75	38.3382716	46.8522835	0	132.0428386
Radiation	79	8.75	13.72008439	18.48971358	0	50.69951155
HD-MTX+ rituximab	40	25.4	61.445	56.67639946	0	174.7977989
Transplant	32	25.4	59.8728125	55.18110317	0	170.2350188
HD-MTX+ transplant	12	25.4	53.7	51.05744251	0	155.814885
Rituximab + transplant	11	20.5	61.78181818	59.61165993	0	181.005138
Corticosteroids	6	13	13.66666667	6.187545094	1.291576479	26.04175685
HD-MTX+ rituximab + transplant	5	79.25	90.5	67.8481761	0	226.1963522

Kruskal-Wallis chi-squared =69.849	df =8	p value =5.267e-12

Pair	Z score	Bonferroni adjusted p value
HD-MTX+ rituximab - Radiation	6.35409211	3.7739E-09
Radiation - Transplant	-5.69482547	2.22297E-07
HD-MTX - Radiation	4.89382139	1.78014E-05
Radiation - Rituximab & R-CHOP	-4.38362727	0.000210095
HD-MTX+ transplant - Radiation	4.03225436	0.000994398
Radiation - Rituximab + transplant	-3.66143811	0.004514462
HD-MTX+ rituximab + transplant - Radiation	3.38964744	0.01259686
HD-MTX+ rituximab - Rituximab & R-CHOP	3.31940784	0.01623754
Rituximab & R-CHOP - Transplant	-2.84304368	0.0804329
HD-MTX - HD-MTX+ rituximab	-2.37627711	0.3147898
Corticosteroids - HD-MTX+ rituximab	-2.2445939	0.4462959
Corticosteroids - HD-MTX+ rituximab + transplant	-2.16793809	0.5429412
Corticosteroids - Transplant	-2.11952381	0.612832
HD-MTX+ transplant - Rituximab & R-CHOP	2.04899789	0.7283218
HD-MTX+ rituximab + transplant - Rituximab & R-CHOP	2.04590106	0.7337899
HD-MTX - Transplant	-2.00899026	0.801687
Corticosteroids - HD-MTX+ transplant	-1.99782643	0.8232387
Corticosteroids - HD-MTX	-1.23720922	1
HD-MTX - HD-MTX+ rituximab + transplant	-1.71283319	1
HD-MTX+ rituximab - HD-MTX+ rituximab + transplant	-0.69585722	1
HD-MTX - HD-MTX+ transplant	-1.5370861	1
HD-MTX+ rituximab - HD-MTX+ transplant	-0.04932722	1
HD-MTX+ rituximab + transplant - HD-MTX+ transplant	0.58959985	1
Corticosteroids - Radiation	0.5912616	1
Corticosteroids - Rituximab & R-CHOP	-0.90846787	1
HD-MTX - Rituximab & R-CHOP	1.00803312	1
Corticosteroids - Rituximab + transplant	-1.8283826	1
HD-MTX - Rituximab + transplant	-1.25875084	1
HD-MTX+ rituximab - Rituximab + transplant	0.16078029	1
HD-MTX+ rituximab + transplant - Rituximab + transplant	0.71346128	1
HD-MTX+ transplant - Rituximab + transplant	0.17002852	1
Rituximab & R-CHOP - Rituximab + transplant	-1.74329176	1
HD-MTX+ rituximab - Transplant	0.16759049	1
HD-MTX+ rituximab + transplant - Transplant	0.76904446	1
HD-MTX+ transplant - Transplant	0.16538519	1
Rituximab + transplant - Transplant	-0.04289046	1

Supplemental Table 6: Analysis of B-cell histologies on (A) OS, (B) treatment 1 to progression, (C) symptom onset to diagnosis, and (D) symptom onset to death periods.

6A

Overall Survival

Types of B-cell lymphoma	N	Median (months)	Mean (months)	Standard deviation	Lower bound	Upper bound
Diffuse large B-cell	250	10	20.11056	7.108532653	5.891494694	34.32962531
Unspecified B-cell	15	5	22.88666667	39.93288775	0	102.7524422
Follicular	13	10	25.44769231	2.683281573	20.08112916	30.81425545
IVL	12	9.4	10.2	#REF!	#REF!	#REF!
Burkitt	11	2.28	3.694545455	2.892092668	0	9.47873079
Lymphoplasmacytic	11	18	25.58181818	25.86135193	0	77.30452205
High-grade B-cell	9	4	8.088888889	10.10673985	0	28.3029686
CLL/SLL	8	17	38.75	36.44950846	0	111.6490169
Mantle cell	8	7.45	23.05	46.7232582	0	116.4965164
Marginal zone	7	25.4	36.94285714	47.15643672	0	131.2557306
Low-grade B-cell	6	81.7	81.7	61.67355998	0	205.04712
Intermediate & triple-hit B-cell	2	3	3	4.242640687	0	11.48528137
MALT	1	0	0	N/A	N/A	N/A
Bacute lymphoblastic leukemia	1	27	27	N/A	N/A	N/A
Plasma cell leukemia	1	38	38	N/A	N/A	N/A
N/A = Not applicable						
Kruskal-Wallis chi-squared = 36.033	df = 14	p value = 0.01032				

Pair	Z score	Bonferroni adjusted p value
Burkitt vs. Low-grade B-cell	-4.09745554	0.002193011
Low-grade B-cell vs. Unspecified B-cell	3.57577872	0.018332314
High-grade B-cell vs. Low-grade B-cell	-3.20065111	0.07198666
IVL vs. Low-grade B-cell	-3.09932628	0.101829675
Diffuse large B-cell vs. Low-grade B-cell	-3.08267462	0.127258326
Intermediate & triple-hit B-cell vs. Low-grade B-cell	-2.75450749	0.308597859
Burkitt vs. Marginal zone	-2.72324958	0.33937681
Burkitt vs. Lymphoplasmacytic	-2.71879582	0.343980208
Low-grade B-cell vs. Mantle cell	2.7166637	0.346208768
Burkitt vs. Diffuse large B-cell	-2.68340222	0.382605739
Low-grade B-cell vs. MALT	2.62323112	0.457275872
Burkitt vs. Follicular	-2.46073783	0.727921129
Bacute lymphoblastic leukemia vs. Burkitt	1.85849489	1
Bacute lymphoblastic leukemia vs. CLL/SLL	0.82823276	1
Burkitt vs. CLL/SLL	-2.28696396	1
Bacute lymphoblastic leukemia vs. Diffuse large B-ce	1.11222809	1
CLL/SLL vs. Diffuse large B-cell	0.6570138	1
Bacute lymphoblastic leukemia vs. Follicular	0.89909614	1
CLL/SLL vs. Follicular	0.12142343	1
Diffuse large B-cell vs. Follicular	-0.6377273	1
Bacute lymphoblastic leukemia vs. High-grade B-cell	1.46902511	1
Burkitt vs. High-grade B-cell	-0.8735826	1
CLL/SLL vs. High-grade B-cell	1.37887934	1
Diffuse large B-cell vs. High-grade B-cell	1.27929067	1
Follicular vs. High-grade B-cell	1.41930677	1
Bacute lymphoblastic leukemia vs. Intermediate & tr	1.72333289	1
Burkitt vs. Intermediate & triple-hit B-cell	0.22061147	1
CLL/SLL vs. Intermediate & triple-hit B-cell	1.55858488	1
Diffuse large B-cell vs. Intermediate & triple-hit B-cel	1.40822763	1
Follicular vs. Intermediate & triple-hit B-cell	1.55039161	1
High-grade B-cell vs. Intermediate & triple-hit B-cell	0.7191107	1
Bacute lymphoblastic leukemia vs. IVL	1.35589505	1
Burkitt vs. IVL	-1.26939241	1
CLL/SLL vs. IVL	1.16727742	1
Diffuse large B-cell vs. IVL	1.00435789	1
Follicular vs. IVL	1.19460356	1
High-grade B-cell vs. IVL	-0.31120411	1
Intermediate & triple-hit B-cell vs. IVL	-0.91570704	1
Bacute lymphoblastic leukemia vs. Low-grade B-cell	-0.12813612	1
CLL/SLL vs. Low-grade B-cell	-1.88288909	1
Follicular vs. Low-grade B-cell	-2.17089063	1
Bacute lymphoblastic leukemia vs. Lymphoplasmacyt	0.74855115	1
CLL/SLL vs. Lymphoplasmacytic	-0.20797419	1
Diffuse large B-cell vs. Lymphoplasmacytic	-1.07965951	1
Follicular vs. Lymphoplasmacytic	-0.36907458	1
High-grade B-cell vs. Lymphoplasmacytic	-1.70569358	1
Intermediate & triple-hit B-cell vs. Lymphoplasmacyt	-1.72862804	1
IVL vs. Lymphoplasmacytic	-1.50787882	1
Low-grade B-cell vs. Lymphoplasmacytic	1.81321016	1
Bacute lymphoblastic leukemia vs. MALT	1.90566028	1
Burkitt vs. MALT	0.72178133	1
CLL/SLL vs. MALT	1.71264761	1
Diffuse large B-cell vs. MALT	1.57740862	1
Follicular vs. MALT	1.69788107	1
High-grade B-cell vs. MALT	1.08768645	1
Intermediate & triple-hit B-cell vs. MALT	0.47713406	1
IVL vs. MALT	1.25338739	1
Lymphoplasmacytic vs. MALT	1.83172507	1
Bacute lymphoblastic leukemia vs. Mantle cell	1.25277001	1
Burkitt vs. Mantle cell	-1.31789072	1
CLL/SLL vs. Mantle cell	0.90057951	1
Diffuse large B-cell vs. Mantle cell	0.59669655	1
Follicular vs. Mantle cell	0.88064894	1
High-grade B-cell vs. Mantle cell	-0.45219067	1
Intermediate & triple-hit B-cell vs. Mantle cell	-0.96900849	1
IVL vs. Mantle cell	-0.18074199	1
Lymphoplasmacytic vs. Mantle cell	1.17704743	1
MALT vs. Mantle cell	-1.28811086	1
Bacute lymphoblastic leukemia vs. Marginal zone	0.5841292	1
CLL/SLL vs. Marginal zone	-0.49079999	1
Diffuse large B-cell vs. Marginal zone	-1.27861452	1
Follicular vs. Marginal zone	-0.65821463	1
High-grade B-cell vs. Marginal zone	-1.83356132	1
Intermediate & triple-hit B-cell vs. Marginal zone	-1.85359555	1
IVL vs. Marginal zone	-1.6543491	1
Low-grade B-cell vs. Marginal zone	1.37119631	1
Lymphoplasmacytic vs. Marginal zone	-0.32549705	1
MALT vs. Marginal zone	-1.9368224	1
Mantle cell vs. Marginal zone	-1.36084245	1
Bacute lymphoblastic leukemia vs. Plasma cell leuke	-0.07883616	1
Burkitt vs. Plasma cell leukemia	-1.96523956	1
CLL/SLL vs. Plasma cell leukemia	-0.93334764	1
Diffuse large B-cell vs. Plasma cell leukemia	-1.22349694	1
Follicular vs. Plasma cell leukemia	-1.00653171	1
High-grade B-cell vs. Plasma cell leukemia	-1.57479491	1
Intermediate & triple-hit B-cell vs. Plasma cell leuke	-1.81436505	1
IVL vs. Plasma cell leukemia	-1.4630123	1
Low-grade B-cell vs. Plasma cell leukemia	0.02491536	1
Lymphoplasmacytic vs. Plasma cell leukemia	-0.85529581	1
MALT vs. Plasma cell leukemia	-1.98449644	1
Mantle cell vs. Plasma cell leukemia	-1.35788489	1
Marginal zone vs. Plasma cell leukemia	-0.68841963	1
Bacute lymphoblastic leukemia vs. Unspecified B-cel	1.35425115	1
Burkitt vs. Unspecified B-cell	-1.83739365	1
CLL/SLL vs. Unspecified B-cell	1.31630843	1
Diffuse large B-cell vs. Unspecified B-cell	2.1558281	1
Follicular vs. Unspecified B-cell	1.45766185	1
High-grade B-cell vs. Unspecified B-cell	-0.54287272	1
Intermediate & triple-hit B-cell vs. Unspecified B-cell	-1.05165998	1
IVL vs. Unspecified B-cell	-0.16757539	1
Lymphoplasmacytic vs. Unspecified B-cell	1.83013205	1
MALT vs. Unspecified B-cell	-1.32870119	1
Mantle cell vs. Unspecified B-cell	0.08625191	1
Marginal zone vs. Unspecified B-cell	1.8883171	1
Plasma cell leukemia vs. Unspecified B-cell	1.46524347	1

6B

Time from Treatment 1 to Progression						
**Types of B-cell lymphoma**	**N**	**Median (months)**	**Mean (months)**	**andard deviatio**	**Lower bound**	**Upper bound**
Unspecified B-cell	62	16.5	19.16807018	16.83790912	0	52.84388841
High-grade B-cell	6	11	11	0	11	11
B acute lymphoblastic leukemia	6	9.4	9.4	0	9.4	9.4
Mantle cell	3	12	14.6	5.392587505	3.81482499	25.38517501
Burkitt	3	11	8.333333333	5.507570547	0	19.34847443
MALT	2	14	14	18.38477631	0	50.76955262
Diffuse large B-cell	2	28.4	28.4	10.74802307	6.903953852	49.89604615
CLL/SLL	2	10.2	10.2	1.13137085	7.9372583	12.4627417
Marginal zone	2	2.48	2.48	2.093036072	0	6.666072145
IVL	1	7	7	N/A	N/A	N/A
Follicular	1	1	1	N/A	N/A	N/A
Lymphoplasmacytic	0	N/A	N/A	N/A	N/A	N/A
Low-grade B-cell	0	N/A	N/A	N/A	N/A	N/A
Intermediate & triple-hit B-cell	0	N/A	N/A	N/A	N/A	N/A
Plasma cell leukemia	0	N/A	N/A	N/A	N/A	N/A
N/A = Not applicable						
**Kruskal-Wallis chi-squared = 9.9899**	**df = 10**	**p value = 0.4414**				

**Pair**	**Z score**	**Bonferroni adjusted p value**
Diffuse large B-cell - Marginal zone	2.1866359	0.7911507
B acute lymphoblastic leukemia - Burkitt	0.09162578	1
B acute lymphoblastic leukemia - CLL/SLL	-0.19044061	1
Burkitt - CLL/SLL	-0.24130829	1
B acute lymphoblastic leukemia - Diffuse large B-cell	-1.38069443	1
Burkitt - Diffuse large B-cell	-1.30590367	1
CLL/SLL - Diffuse large B-cell	-0.97183818	1
B acute lymphoblastic leukemia - Follicular	1.20566141	1
Burkitt - Follicular	1.07168387	1
CLL/SLL - Follicular	1.19025382	1
Diffuse large B-cell - Follicular	1.98375637	1
B acute lymphoblastic leukemia - High-grade B-cell	-0.53864739	1
Burkitt - High-grade B-cell	-0.53142954	1
CLL/SLL - High-grade B-cell	-0.19044061	1
Diffuse large B-cell - High-grade B-cell	0.99981321	1
Follicular - High-grade B-cell	-1.49358055	1
B acute lymphoblastic leukemia - IVL	0.14395957	1
Burkitt - IVL	0.07855274	1
CLL/SLL - IVL	0.25392082	1
Diffuse large B-cell - IVL	1.04742336	1
Follicular - IVL	-0.81088817	1
High-grade B-cell - IVL	0.43187871	1
B acute lymphoblastic leukemia - MALT	0.22614823	1
Burkitt - MALT	0.1313001	1
CLL/SLL - MALT	0.34014336	1
Diffuse large B-cell - MALT	1.31198154	1
Follicular - MALT	-0.91252793	1
High-grade B-cell - MALT	0.60702945	1
IVL - MALT	0.02380508	1
B acute lymphoblastic leukemia - Mantle cell	-0.76965657	1
Burkitt - Mantle cell	-0.7458924	1
CLL/SLL - Mantle cell	-0.42583815	1
Diffuse large B-cell - Mantle cell	0.63875723	1
Follicular - Mantle cell	-1.59910944	1
High-grade B-cell - Mantle cell	-0.32985282	1
IVL - Mantle cell	-0.60597832	1
MALT - Mantle cell	-0.79844654	1
B acute lymphoblastic leukemia - Marginal zone	1.29737667	1
Burkitt - Marginal zone	1.08943594	1
CLL/SLL - Marginal zone	1.21479772	1
Follicular - Marginal zone	-0.19837564	1
High-grade B-cell - Marginal zone	1.67825789	1
IVL - Marginal zone	0.73795737	1
MALT - Marginal zone	0.87465436	1
Mantle cell - Marginal zone	1.75658238	1
B acute lymphoblastic leukemia - Unspecified B-cell	-0.91728984	1
Burkitt - Unspecified B-cell	-0.77301919	1
CLL/SLL - Unspecified B-cell	-0.32945801	1
Diffuse large B-cell - Unspecified B-cell	1.02328348	1
Follicular - Unspecified B-cell	-1.6809451	1
High-grade B-cell - Unspecified B-cell	-0.18991053	1
IVL - Unspecified B-cell	-0.5433138	1
MALT - Unspecified B-cell	-0.80291753	1
Mantle cell - Unspecified B-cell	0.25720168	1
Marginal zone - Unspecified B-cell	-2.02038487	1
Lymphoplasmacytic - MALT	1.00783217	1
B acute lymphoblastic leukemia - Mantle cell	-0.40154821	1

6C

Time from Symptom Onset to Diagnosis						
Types of B-cell lymphoma	N	Median (months)	Mean (months)	tandard deviatio	lower bound	Upper bound
Unspecified B-cell	76	3	9.736842105	18.9004251	0	47.53769251
Diffuse large B-cell	7	3	5.214285714	5.801272438	0	16.81683059
Lymphoplasmacytic	3	36	32	30.19933774	0	92.39867548
Follicular	2	1.75	1.75	1.767766953	0	5.285533906
Burkitt	2	2.5	2.5	2.121320344	0	6.742640687
Mantle cell	2	7	7	1.414213562	4.171572875	9.828427125
Marginal zone	1	5	5	N/A	N/A	N/A
CLL/SLL	1	1	1	N/A	N/A	N/A
IVL	0	N/A	N/A	N/A	N/A	N/A
Low-grade B-cell	0	N/A	N/A	N/A	N/A	N/A
High-grade B-cell	0	N/A	N/A	N/A	N/A	N/A
MALT	0	N/A	N/A	N/A	N/A	N/A
Intermediate & triple-hit B-cell	0	N/A	N/A	N/A	N/A	N/A
Bacute lymphoblastic leukemia	0	N/A	N/A	N/A	N/A	N/A
Plasma cell leukemia	0	N/A	N/A	N/A	N/A	N/A
N/A = Not applicable						

Kruskal-Wallis chi-squared = 4.9476	df = 7	p value = 0.6664

Pair	Z score	Bonferroni adjusted p value
Burkitt vs. CLL/SLL	0.62308542	1
Burkitt vs. Diffuse large B-cell	-0.5651698	1
CLL/SLL vs. Diffuse large B-cell	-1.13771138	1
Burkitt vs. Follicular	0.39535168	1
CLL/SLL vs. Follicular	-0.30028213	1
Diffuse large B-cell vs. Follicular	1.05825997	1
Burkitt vs. Lymphoplasmacytic	-0.90981645	1
CLL/SLL vs. Lymphoplasmacytic	-1.38015495	1
Diffuse large B-cell vs. Lymphoplasmacytic	-0.54690607	1
Follicular vs. Lymphoplasmacytic	-1.34290252	1
Burkitt vs. Mantle cell	-1.14927813	1
CLL/SLL vs. Mantle cell	-1.56146708	1
Diffuse large B-cell vs. Mantle cell	-0.86823186	1
Follicular vs. Mantle cell	-1.5446298	1
Lymphoplasmacytic vs. Mantle cell	-0.34915465	1
Burkitt vs. Marginal zone	-0.74319828	1
CLL/SLL vs. Marginal zone	-1.18323639	1
Diffuse large B-cell vs. Marginal zone	-0.42756324	1
Follicular vs. Marginal zone	-1.06600157	1
Lymphoplasmacytic vs. Marginal zone	-0.06900775	1
Mantle cell vs. Marginal zone	0.19518339	1
Burkitt vs. Unspecified B-cell	-0.48468315	1
CLL/SLL vs. Unspecified B-cell	-1.10309019	1
Diffuse large B-cell vs. Unspecified B-cell	0.26821357	1
Follicular vs. Unspecified B-cell	-1.08658021	1
Lymphoplasmacytic vs. Unspecified B-cell	0.82112341	1
Mantle cell vs. Unspecified B-cell	1.11966875	1
Marginal zone vs. Unspecified B-cell	0.55935735	1
Follicular vs. IVL	0	1
High-grade B-cell vs. IVL	0.56287063	1
Intermediate & triple-hit B-cell vs. IVL	0	1
Bacute lymphoblastic leukemia vs. Low-grade B-cell	0	1
Burkitt vs. low-grade B-cell	1.06242499	1
CLL/SLL vs. low-grade B-cell	1.39085328	1
Diffuse large B-cell vs. low-grade B-cell	1.83086163	1
Follicular vs. Low-grade B-cell	0	1
High-grade B-cell vs. low-grade B-cell	0.51847827	1
Intermediate & triple-hit B-cell vs. low-grade B-cell	0	1
IVL vs. Low-grade B-cell	0	1
Bacute lymphoblastic leukemia vs. Lymphoplasmacytic	-0.18607285	1
Burkitt vs. Lymphoplasmacytic	0.6041749	1
CLL/SLL vs. Lymphoplasmacytic	0.97263709	1
Diffuse large B-cell vs. Lymphoplasmacytic	1.26552607	1
Follicular vs. Lymphoplasmacytic	-0.50006575	1
High-grade B-cell vs. Lymphoplasmacytic	0.07099673	1
Intermediate & triple-hit B-cell vs. Lymphoplasmacytic	-0.30273651	1
IVL vs. Lymphoplasmacytic	-0.54049277	1
Low-grade B-cell vs. Lymphoplasmacytic	-0.48959004	1
Bacute lymphoblastic leukemia vs. MALT	0	1
Burkitt vs. MALT	0.80821407	1
CLL/SLL vs. MALT	1.08688915	1
Diffuse large B-cell vs. MALT	1.19350926	1
Follicular vs. MALT	0	1
High-grade B-cell vs. MALT	0.40531311	1
Intermediate & triple-hit B-cell vs. MALT	0	1
IVL vs. MALT	0	1
Low-grade B-cell vs. MALT	0	1
Lymphoplasmacytic vs. MALT	0.36968926	1
Bacute lymphoblastic leukemia vs. Mantle cell	-0.37225118	1
Burkitt vs. Mantle cell	0.09457192	1
CLL/SLL vs. Mantle cell	0.48546196	1
Diffuse large B-cell vs. Mantle cell	0.51131338	1
Follicular vs. Mantle cell	-0.96785307	1
High-grade B-cell vs. Mantle cell	-0.38641305	1
Intermediate & triple-hit B-cell vs. Mantle cell	-0.60023699	1
IVL vs. Mantle cell	-1.08964097	1
Low-grade B-cell vs. Mantle cell	-0.94905802	1
Lymphoplasmacytic vs. Mantle cell	-0.49529401	1
MALT vs. Mantle cell	-0.72789427	1
Bacute lymphoblastic leukemia vs. Marginal zone	-0.39027686	1
Burkitt vs. Marginal zone	0.04770516	1
CLL/SLL vs. Marginal zone	0.44164408	1
Diffuse large B-cell vs. Marginal zone	0.44741634	1
Follicular vs. Marginal zone	-1.01471983	1
High-grade B-cell vs. Marginal zone	-0.43023098	1
Intermediate & triple-hit B-cell vs. Marginal zone	-0.62930252	1
IVL vs. Marginal zone	-1.08998394	1
Low-grade B-cell vs. Marginal zone	-0.99501466	1
Lymphoplasmacytic vs. Marginal zone	-0.54298546	1
MALT vs. Marginal zone	-0.7631414	1
Mantle cell vs. Marginal zone	-0.04595664	1
Bacute lymphoblastic leukemia vs. Plasma cell leukemia	0	1
Burkitt vs. Plasma cell leukemia	0.40983934	1
CLL/SLL vs. Plasma cell leukemia	0.56760935	1
Diffuse large B-cell vs. Plasma cell leukemia	0.53681255	1
Follicular vs. Plasma cell leukemia	0	1
High-grade B-cell vs. Plasma cell leukemia	0.21166787	1
Intermediate & triple-hit B-cell vs. Plasma cell leukemia	0	1
IVL vs. Plasma cell leukemia	0	1
Low-grade B-cell vs. Plasma cell leukemia	0	1
Lymphoplasmacytic vs. Plasma cell leukemia	0.18607285	1
MALT vs. Plasma cell leukemia	0	1
Mantle cell vs. Plasma cell leukemia	0.37225118	1
Marginal zone vs. Plasma cell leukemia	0.39027686	1
Bacute lymphoblastic leukemia vs. Unspecified B-cell	-0.97138878	1
Burkitt vs. Unspecified B-cell	-1.57344576	1
CLL/SLL vs. Unspecified B-cell	-1.0210141	1
Diffuse large B-cell vs. Unspecified B-cell	-1.90462918	1
High-grade B-cell vs. Unspecified B-cell	-1.96753931	1
Intermediate & triple-hit B-cell vs. Unspecified B-cell	-1.6003717	1
Lymphoplasmacytic vs. Unspecified B-cell	-2.2801577	1
MALT vs. Unspecified B-cell	-1.97419918	1
Mantle cell vs. Unspecified B-cell	-1.63829309	1
Marginal zone vs. Unspecified B-cell	-1.58795011	1
Plasma cell leukemia vs. Unspecified B-cell	-0.97138878	1

6D

Time from Symptom Onset to Death						
Types of B-cell lymphoma	N	Median (months)	Mean (months)	Standard deviatio	Lower bound	Upper bound
Diffuse large B-cell	263	10	21.93019011	32.8752773	0	87.68074471
Unspecified B-cell	17	4	22.34117647	39.32219569	0	100.9855678
Follicular	13	10	25.44769231	38.51564491	0	102.4789821
Burkitt	12	3.14	4.553333333	4.011821924	0	12.57697718
IVL	12	9.4	10.2	11.03374988	0	32.26749976
Lymphoplasmacytic	11	18	26.03636364	28.55406355	0	83.14449074
CLL/SLL	9	10	45.11111111	58.89491583	0	162.9009428
High-grade B-cell	9	5	8.2	9.004026877	0	26.20805375
Mantle cell	8	8.95	23.4875	46.54308104	0	116.5736621
Marginal zone	8	18.7	32.95	45.08591798	0	123.121836
Low-grade B-cell	6	81.7	81.7	61.67355998	0	205.04712
Intermediate & triple-hit B-cell	2	3	3	4.242640687	0	11.48528137
MALT	1	0	0	N/A	N/A	N/A
B acute lymphoblastic leukemia	1	27	27	N/A	N/A	N/A
Plasma cell leukemia	1	38	38	N/A	N/A	N/A
N/A = Not applicable						
Kruskal-Wallis chi-squared = 35.834	df= 14	p value = 0.01105				

Pair	Z score	Bonferroni adjusted p value
Burkitt vs. Low-grade B-cell	-4.20588886	0.001365305
Low-grade B-cell vs. Unspecified B-cell	3.55434603	0.019893316
High-grade B-cell vs. Low-grade B-cell	-3.37082745	0.039416427
IVL vs. Low-grade B-cell	-3.29489673	0.051690873
Diffuse large B-cell vs. Low-grade B-cell	-2.9749092	0.153864526
Burkitt vs. Diffuse large B-cell	-2.96309145	0.159897188
Intermediate & triple-hit B-cell vs. Low-grade B-cell	-2.85521459	0.225790732
Burkitt vs. Lymphoplasmacytic	-2.8108229	0.259832049
Low-grade B-cell vs. Mantle cell	2.77746459	0.287620245
Low-grade B-cell vs. MALT	2.66782783	0.400802682
Burkitt vs. Marginal zone	-2.53046517	0.598084818
Burkitt vs. Follicular	-2.42893748	0.806045544
B acute lymphoblastic leukemia vs. Burkitt	1.81743257	1
B acute lymphoblastic leukemia vs. CLL/SLL	0.90120157	1
Burkitt vs. CLL/SLL	-2.13556108	1
B acute lymphoblastic leukemia vs. Diffuse large B-cell	1.01504854	1
CLL/SLL vs. Diffuse large B-cell	0.19772511	1
B acute lymphoblastic leukemia vs. Follicular	0.88777939	1
CLL/SLL vs. Follicular	-0.06608796	1
Diffuse large B-cell vs. Follicular	-0.33677162	1
B acute lymphoblastic leukemia vs. High-grade B-cell	1.48470634	1
Burkitt vs. High-grade B-cell	-0.7407179	1
CLL/SLL vs. High-grade B-cell	1.30475633	1
Diffuse large B-cell vs. High-grade B-cell	1.61669492	1
Follicular vs. High-grade B-cell	1.48450763	1
B acute lymphoblastic leukemia vs. Intermediate & triple-hit B-cel	1.73095015	1
Burkitt vs. Intermediate & triple-hit B-cell	0.29895222	1
CLL/SLL vs. Intermediate & triple-hit B-cell	1.49669775	1
Diffuse large B-cell vs. Intermediate & triple-hit B-cell	1.55397424	1
Follicular vs. Intermediate & triple-hit B-cell	1.5781357	1
High-grade B-cell vs. Intermediate & triple-hit B-cell	0.70990008	1
B acute lymphoblastic leukemia vs. IVL	1.37980611	1
Burkitt vs. IVL	-1.11573293	1
CLL/SLL vs. IVL	1.10258311	1
Diffuse large B-cell vs. IVL	1.42001737	1
Follicular vs. IVL	1.28610868	1
High-grade B-cell vs. IVL	-0.29225007	1
Intermediate & triple-hit B-cell vs. IVL	-0.89533655	1
B acute lymphoblastic leukemia vs. Low-grade B-cell	-0.19562638	1
CLL/SLL vs. Low-grade B-cell	-2.20831792	1
Follicular vs. Low-grade B-cell	-2.29479722	1
B acute lymphoblastic leukemia vs. Lymphoplasmacytic	0.68795812	1
CLL/SLL vs. Lymphoplasmacytic	-0.51483484	1
Diffuse large B-cell vs. Lymphoplasmacytic	-0.96970132	1
Follicular vs. Lymphoplasmacytic	-0.49489048	1
High-grade B-cell vs. Lymphoplasmacytic	-1.88327482	1
Intermediate & triple-hit B-cell vs. Lymphoplasmacytic	-1.82909399	1
IVL vs. Lymphoplasmacytic	-1.71911457	1
Low-grade B-cell vs. Lymphoplasmacytic	1.83214629	1
B acute lymphoblastic leukemia vs. MALT	1.88817505	1
Burkitt vs. MALT	0.74809213	1
CLL/SLL vs. MALT	1.63205109	1
Diffuse large B-cell vs. MALT	1.65017207	1
Follicular vs. MALT	1.68536947	1
High-grade B-cell vs. MALT	1.04854632	1
Intermediate & triple-hit B-cell vs. MALT	0.4493266	1
IVL vs. MALT	1.18571859	1
Lymphoplasmacytic vs. MALT	1.86864299	1
B acute lymphoblastic leukemia vs. Mantle cell	1.2149996	1
Burkitt vs. Mantle cell	-1.32097998	1
CLL/SLL vs. Mantle cell	0.69714548	1
Diffuse large B-cell vs. Mantle cell	0.75712802	1
Follicular vs. Mantle cell	0.81763105	1
High-grade B-cell vs. Mantle cell	-0.56865408	1
Intermediate & triple-hit B-cell vs. Mantle cell	-1.05148342	1
IVL vs. Mantle cell	-0.32903811	1
Lymphoplasmacytic vs. Mantle cell	1.22708188	1
MALT vs. Mantle cell	-1.30256714	1
B acute lymphoblastic leukemia vs. Marginal zone	0.69452004	1
CLL/SLL vs. Marginal zone	-0.43896808	1
Diffuse large B-cell vs. Marginal zone	-0.78109294	1
Follicular vs. Marginal zone	-0.41090253	1
High-grade B-cell vs. Marginal zone	-1.7047676	1
Intermediate & triple-hit B-cell vs. Marginal zone	-1.74978008	1
IVL vs. Marginal zone	-1.5325253	1
Low-grade B-cell vs. Marginal zone	1.75526303	1
Lymphoplasmacytic vs. Marginal zone	0.0389552	1
MALT vs. Marginal zone	-1.82904669	1
Mantle cell vs. Marginal zone	-1.10410887	1
B acute lymphoblastic leukemia vs. Plasma cell leukemia	-0.13135131	1
Burkitt vs. Plasma cell leukemia	-1.99590885	1
CLL/SLL vs. Plasma cell leukemia	-1.07742785	1
Diffuse large B-cell vs. Plasma cell leukemia	-1.2004552	1
Follicular vs. Plasma cell leukemia	-1.06678105	1
High-grade B-cell vs. Plasma cell leukemia	-1.66093261	1
Intermediate & triple-hit B-cell vs. Plasma cell leukemia	-1.88262158	1
IVL vs. Plasma cell leukemia	-1.55827739	1
Low-grade B-cell vs. Plasma cell leukemia	0.02364714	1
Lymphoplasmacytic vs. Plasma cell leukemia	-0.86580863	1
MALT vs. Plasma cell leukemia	-2.01952636	1
Mantle cell vs. Plasma cell leukemia	-1.39013468	1
Marginal zone vs. Plasma cell leukemia	-0.86965512	1
B acute lymphoblastic leukemia vs. Unspecified B-cell	1.43490911	1
Burkitt vs. Unspecified B-cell	-1.10104459	1
CLL/SLL vs. Unspecified B-cell	1.27733976	1
Diffuse large B-cell vs. Unspecified B-cell	1.83628406	1
Follicular vs. Unspecified B-cell	1.50694823	1
High-grade B-cell vs. Unspecified B-cell	-0.21470618	1
Intermediate & triple-hit B-cell vs. Unspecified B-cell	-0.86076828	1
IVL vs. Unspecified B-cell	0.10704936	1
Lymphoplasmacytic vs. Unspecified B-cell	1.95879212	1
MALT vs. Unspecified B-cell	-1.16013928	1
Mantle cell vs. Unspecified B-cell	0.43808886	1
Marginal zone vs. Unspecified B-cell	1.72563417	1
Plasma cell leukemia vs. Unspecified B-cell	1.61543422	1

Supplemental Table 7: Analysis of T-cell histologies on (A) OS, (B) treatment 1 to progression, (C) symptom onset to diagnosis, and (D) symptom onset to death periods.

7A

**Overall Survival**						
**Types of T-cell lymphoma**	**N**	**Median (months)**	**Mean (months)**	**tandard deviatic**	**Lower bound**	**Upper bound**
T lymphoblastic lymphoma/leukemia	3	10	11.55333333	4.956060263	1.641212807	21.46545386
Peripheral T-cell	7	10	33.96142857	47.265002	0	128.4914326
Cutaneous T-cell	4	3	5.625	5.618051264	0	16.86110253
Adult T-cell leukemia/lymphoma	2	45.5	45.5	64.34671709	0	174.1934342
Extranodal NK/T-cell* (lymphoma, nasal type)	7	1	5.28	5.29683868	0	15.87367736
ALCL	2	11.65	11.65	10.81873375	0	33.2874675
Unspecified T-cell	15	10	16.40666667	15.69160224	0	47.78987115
Enteropathy-associated T-cell	1	1.3	1.3	N/A	N/A	N/A
N/A = Not applicable						
**Kruskal-Wallis chi-squared = 8.5973**	**df = 7**	**p value = 0.2829**				
**Pair**	**Z score**	**Bonferroni adjusted p value**				
Extranodal NK/T-cell* (lymphoma, nasal type) vs. Peripheral T-cell	-2.29291654	0.3059393				
Extranodal NK/T-cell* (lymphoma, nasal type) vs. Unspecified T-cell	-1.97228658	0.6800766				
Adult T-cell leukemia/lymphoma vs. ALCL	-0.18924944	1				
Adult T-cell leukemia/lymphoma vs. Cutaneous T-cell	0.60701787	1				
ALCL vs. Cutaneous T-cell	0.82554431	1				
Adult T-cell leukemia/lymphoma vs. Enteropathy-associated T-cell	0.87562197	1				
ALCL vs. Enteropathy-associated T-cell	1.03014349	1				
Cutaneous T-cell vs. Enteropathy-associated T-cell	0.48900178	1				
Adult T-cell leukemia/lymphoma vs. Extranodal NK/T-cell* (lymphoma, nasal type)	0.81301126	1				
ALCL vs. Extranodal NK/T-cell* (lymphoma, nasal type)	1.04904678	1				
Cutaneous T-cell vs. Extranodal NK/T-cell* (lymphoma, nasal type)	0.20129177	1				
Enteropathy-associated T-cell vs. Extranodal NK/T-cell* (lymphoma, nasal type)	-0.39339254	1				
Adult T-cell leukemia/lymphoma vs. Peripheral T-cell	-0.71559977	1				
ALCL vs. Peripheral T-cell	-0.47956424	1				
Cutaneous T-cell vs. Peripheral T-cell	-1.75411402	1				
Enteropathy-associated T-cell vs. Peripheral T-cell	-1.53985082	1				
Adult T-cell leukemia/lymphoma vs. T lymphoblastic lymphoma/leukemia	-0.16124296	1				
ALCL vs. T lymphoblastic lymphoma/leukemia	0.04606942	1				
Cutaneous T-cell vs. T lymphoblastic lymphoma/leukemia	-0.88101577	1				
Enteropathy-associated T-cell vs. T lymphoblastic lymphoma/leukemia	-1.0562111	1				
Extranodal NK/T-cell* (lymphoma, nasal type) vs. T lymphoblastic lymphoma/leukemia	-1.15793811	1				
Peripheral T-cell vs. T lymphoblastic lymphoma/leukemia	0.61814741	1				
Adult T-cell leukemia/lymphoma vs. Unspecified T-cell	-0.333342	1				
ALCL vs. Unspecified T-cell	-0.08193882	1				
Cutaneous T-cell vs. Unspecified T-cell	-1.38009643	1				
Enteropathy-associated T-cell vs. Unspecified T-cell	-1.28132257	1				
Peripheral T-cell vs. Unspecified T-cell	0.70526308	1				
T lymphoblastic lymphoma/leukemia vs. Unspecified T-cell	-0.16402218	1				

7B

Time from Treatment 1 to Progression						
**Types of T-cell lymphoma**	**N**	**Median (months)**	**Mean (months)**	**tandard deviatic**	**Lower bound**	**Upper bound**
Unspecified T-cell	3	9.4	8.133333333	5.608327142	0	19.34998762
Peripheral T-cell	1	11	11	N/A	N/A	N/A
Extranodal NK/T-cell* (lymphoma, nasal type)	1	8	8	N/A	N/A	N/A
T lymphoblastic lymphoma/leukemia	0	N/A	N/A	N/A	N/A	N/A
Cutaneous T-cell	0	N/A	N/A	N/A	N/A	N/A
Adult T-cell leukemia/lymphoma	0	N/A	N/A	N/A	N/A	N/A
ALCL	0	N/A	N/A	N/A	N/A	N/A
Enteropathy-associated T-cell	0	N/A	N/A	N/A	N/A	N/A
N/A = Not applicable						
**Kruskal-Wallis chi-squared = 0.8**	**df = 2**	**p value = 0.6703**				
**Pair**	**Z score**	**Bonferroni adjusted p value**				
Extranodal NK/T-cell* (lymphoma, nasal type) vs. Peripheral T-cell	-0.8944272	0.5566401				
Extranodal NK/T-cell* (lymphoma, nasal type) vs. Unspecified T-cell	-0.5477226	0.8758236				
Peripheral T-cell vs. Unspecified T-cell	0.5477226	0.8758236				

7C

Time from Symptom Onset to Diagnosis						
**Types of T-cell lymphoma**	**N**	**Median (months)**	**Mean (months)**	**tandard deviatio**	**Lower bound**	**Upper bound**
Extranodal NK/T-cell* (lymphoma, nasal type)	5	2	2.466666667	2.216228027	0	6.89912272
Peripheral T-cell	2	6.125	6.125	8.308504679	0	22.74200936
Unspecified T-cell	2	4	4	2.828427125	0	9.656854249
T lymphoblastic lymphoma/leukemia	1	0.9	0.9	N/A	N/A	N/A
Cutaneous T-cell	1	2	2	N/A	N/A	N/A
Adult T-cell leukemia/lymphoma	1	6	6	N/A	N/A	N/A
Enteropathy-associated T-cell	1	0	0	N/A	N/A	N/A
ALCL	0	N/A	N/A	N/A	N/A	N/A
N/A = Not applicable						
**Kruskal-Wallis chi-squared = 4.6854**	**df = 6**	**p value = 0.5847**				
**Pair**	**Z score**	**Bonferroni adjusted p value**				
Adult T-cell leukemia/lymphoma vs. Enteropathy-associated T-cell	1.8359702	0.6968017				
Enteropathy-associated T-cell vs. Unspecified T-cell	-1.6959966	0.9438085				
Adult T-cell leukemia/lymphoma vs. Cutaneous T-cell	0.7343881	1				
Cutaneous T-cell vs. Enteropathy-associated T-cell	1.1015821	1				
Adult T-cell leukemia/lymphoma vs. Extranodal NK/T-cell* (lymphoma, nasal type)	0.9480909	1				
Cutaneous T-cell vs. Extranodal NK/T-cell* (lymphoma, nasal type)	0	1				
Enteropathy-associated T-cell vs. Extranodal NK/T-cell* (lymphoma, nasal type)	-1.4221364	1				
Adult T-cell leukemia/lymphoma vs. Peripheral T-cell	0.7419985	1				
Cutaneous T-cell vs. Peripheral T-cell	-0.1059998	1				
Enteropathy-associated T-cell vs. Peripheral T-cell	-1.3779972	1				
Extranodal NK/T-cell* (lymphoma, nasal type) vs. Peripheral T-cell	-0.1551678	1				
Adult T-cell leukemia/lymphoma vs. T lymphoblastic lymphoma/leukemia	1.2851791	1				
Cutaneous T-cell vs. T lymphoblastic lymphoma/leukemia	0.5507911	1				
Enteropathy-associated T-cell vs. T lymphoblastic lymphoma/leukemia	-0.5507911	1				
Extranodal NK/T-cell* (lymphoma, nasal type) vs. T lymphoblastic lymphoma/leukemia	0.7110682	1				
Peripheral T-cell vs. T lymphoblastic lymphoma/leukemia	0.7419985	1				
Adult T-cell leukemia/lymphoma vs. Unspecified T-cell	0.4239992	1				
Cutaneous T-cell vs. Unspecified T-cell	-0.4239992	1				
Extranodal NK/T-cell* (lymphoma, nasal type) vs. Unspecified T-cell	-0.6206712	1				
Peripheral T-cell vs. Unspecified T-cell	-0.3894681	1				
T lymphoblastic lymphoma/leukemia vs. Unspecified T-cell	-1.0599979	1				

7D

Time from Symptom Onset to Death

**Types of T-cell lymphoma**	**N**	**Median (months)**	**Mean (months)**	**tandard deviatio**	**Lower bound**	**Upper bound**
T lymphoblastic lymphoma/leukemia	3	10	11.85333333	5.461184243	0.930964848	22.77570182
Peripheral T-cell	7	19	35.71142857	47.02980878	0	129.7710461
Cutaneous T-cell	4	4.25	6.125	5.390964663	0	16.90692933
Adult T-cell leukemia/lymphoma	2	48.5	48.5	60.1040764	0	168.7081528
Extranodal NK/T-cell* (lymphoma, nasal type)	7	5.996666667	7.041904762	6.530582714	0	20.10307019
ALCL	2	11.65	11.65	10.81873375	0	33.2874675
Unspecified T-cell	18	10	14.83888889	14.78946918	0	44.41782725
Enteropathy-associated T-cell	1	1.3	1.3	N/A	N/A	N/A

N/A = Not applicable

Kruskal-Wallis chi-squared =8.489 | **df = 7** | **p value = 0.2915**

**Pair**	**Z score**	**Bonferroni adjusted p value**
Extranodal NK/T-cell* (lymphoma, nasal type) - Peripheral T-cell	-2.08303345	0.5214744
Cutaneous T-cell - Peripheral T-cell	-1.84559896	0.9093064
Enteropathy-associated T-cell - Peripheral T-cell	-1.80564457	0.9936343
Adult T-cell leukemia/lymphoma - ALCL	0.43082398	1
Adult T-cell leukemia/lymphoma - Cutaneous T-cell	1.2776003	1
ALCL - Cutaneous T-cell	0.78012762	1
Adult T-cell leukemia/lymphoma - Enteropathy-associated T-cell	1.53498026	1
ALCL - Enteropathy-associated T-cell	1.18321395	1
Cutaneous T-cell - Enteropathy-associated T-cell	0.69186168	1
Adult T-cell leukemia/lymphoma - Extranodal NK/T-cell* (lymphoma, nasal type)	1.32588394	1
ALCL - Extranodal NK/T-cell* (lymphoma, nasal type)	0.78855203	1
Cutaneous T-cell - Extranodal NK/T-cell* (lymphoma, nasal type)	-0.06918207	1
Enteropathy-associated T-cell - Extranodal NK/T-cell* (lymphoma, nasal type)	-0.76412785	1
Adult T-cell leukemia/lymphoma - Peripheral T-cell	-0.06280503	1
ALCL - Peripheral T-cell	-0.60013694	1
Adult T-cell leukemia/lymphoma - T lymphoblastic lymphoma/leukemia	0.38613602	1
ALCL - T lymphoblastic lymphoma/leukemia	-0.085808	1
Cutaneous T-cell - T lymphoblastic lymphoma/leukemia	-0.98714175	1
Enteropathy-associated T-cell - T lymphoblastic lymphoma/leukemia	-1.32282509	1
Extranodal NK/T-cell* (lymphoma, nasal type) - T lymphoblastic lymphoma/leukemia	-1.02972798	1
Peripheral T-cell - T lymphoblastic lymphoma/leukemia	0.58378279	1
Adult T-cell leukemia/lymphoma - Unspecified T-cell	0.52838382	1
ALCL - Unspecified T-cell	-0.04962721	1
Cutaneous T-cell - Unspecified T-cell	-1.2891424	1
Enteropathy-associated T-cell - Unspecified T-cell	-1.446488	1
Extranodal NK/T-cell* (lymphoma, nasal type) - Unspecified T-cell	-1.50243585	1
Peripheral T-cell - Unspecified T-cell	0.99720429	1
T lymphoblastic lymphoma/leukemia - Unspecified T-cell	0.0662942	1

Supplemental Table 8: Analysis of peripheral nerve involvements on (A) OS, (B) treatment 1 to progression, (C) symptom onset to diagnosis, and (D) symptom onset to death periods.

8A

Overall Survival							
Category & Types of Peripheral Nerves		N	Median (months)	Mean (months)	Standard deviation	Lower bound	Upper bound
Cranial nerves	Cranial nerves	106	10	24.7682075	40.88375262	0	91.76750524
Neural plexus	Brachial plexus	99	7	20.3367677	33.9720242	0	74.9440484
	Lumbosacral plexus	114	8.52	24.6679825	40.34197924	0	89.20395848
	Unspecified plexus	114	8.52	24.6679825	40.34197924	0	89.20395848
Spinal nerves	Cervical nerve roots	42	4	10.7280952	20.83334759	0	45.66669518
	Thoracic nerve roots	29	5	10.9420690	19.43490773	0	43.86981546
	Lumbar & sacral nerve roots	57	7.56	16.6591228	21.0904757	0	48.7409514
	Unspecified spinal nerve roots	33	10	17.1715152	31.4554133	0	72.9108266
Peripheral nerves	Distal upper extremity nerves	27	5	9.7814815	18.06786431	0	41.13572862
	Distal lower extremity nerves	114	6.35	21.2475439	36.34545657	0	79.04091314
	Unspecified peripheral nerves	74	10	14.3059459	18.40349921	0	46.80699843

Kruskal-Wallis chi-squared = 49.824 | df = 10 | p value = 0.00602

Pair	Z score	Bonferroni adjusted p value
Cervical nerve roots vs. Unspecified peripheral nerves	-3.72466713	0.00537826
Distal upper extremities nerves vs. Unspecified peripheral nerves	-3.18487216	0.03982494
Cervical nerve roots vs. Cranial nerves	-2.75961929	0.15913908
Thoracic nerve roots vs. Unspecified peripheral nerves	-2.73253128	0.17283668
Cervical nerve roots vs. Lumbosacral plexus	-2.70501651	0.18782773
Cervical nerve roots vs. Unspecified plexus	-2.70501651	0.18782773
Cervical nerve roots vs. Unspecified spinal nerve roots	-2.64838488	0.22241282
Cervical nerve roots vs. Lumbar & sacral nerve roots	-2.40797836	0.44113112
Distal upper extremities nerves vs. Unspecified spinal nerve roots	-2.36055961	0.5018031
Distal lower extremity nerves vs. Unspecified peripheral nerves	-2.32158588	0.55701923
Cranial nerves vs. Distal upper extremities nerves	2.31779462	0.56266336
Brachial plexus vs. Cervical nerve roots	2.30035881	0.58926726
Distal upper extremities nerves vs. Lumbosacral plexus	-2.26490746	0.64675487
Distal upper extremities nerves vs. Unspecified plexus	-2.26490746	0.64675487
Brachial plexus vs. Cranial nerves	-0.569152	1
Brachial plexus vs. Distal lower extremity nerves	0.36836457	1
Cervical nerve roots vs. Distal lower extremity nerves	-2.06645094	1
Cranial nerves vs. Distal lower extremity nerves	0.96461306	1
Brachial plexus vs. Distal upper extremities nerves	1.93494226	1
Cervical nerve roots vs. Distal upper extremities nerves	-0.01421201	1
Distal lower extremity nerves vs. Distal upper extremities nerves	1.72637054	1
Brachial plexus vs. Lumbar & sacral nerve roots	-0.39736229	1
Cranial nerves vs. Lumbar & sacral nerve roots	0.08207385	1
Distal lower extremity nerves vs. Lumbar & sacral nerve roots	-0.71922628	1
Distal upper extremities nerves vs. Lumbar & sacral nerve roots	-2.08097748	1
Brachial Plexus vs. Lumbosacral plexus	-0.47065279	1
Cranial nerves vs. Lumbosacral plexus	0.11036353	1
Distal lower extremity nerves vs. Lumbosacral plexus	-0.87021819	1
Lumbar & sacral nerve roots vs. Lumbosacral plexus	0.00869611	1
Brachial plexus vs. Thoracic nerve roots	1.43347477	1
Cervical nerve roots vs. Thoracic nerve roots	-0.50087593	1
Cranial nerves vs. Thoracic nerve roots	1.82391454	1
Distal lower extremity nerves vs. Thoracic nerve roots	1.21200941	1
Distal upper extremities nerves vs. Thoracic nerve roots	-0.43908295	1
Lumbar & sacral nerve roots vs. Thoracic nerve roots	1.61663893	1
Lumbosacral plexus vs. Thoracic nerve roots	1.76621913	1
Brachial plexus vs. Unspecified Peripheral nerves	-1.92599532	1
Cranial nerves vs. Unspecified Peripheral nerves	-1.4286572	1
Lumbar & sacral nerve roots vs. Unspecified Peripheral nerves	-1.30453549	1
Lumbosacral plexus vs. Unspecified Peripheral nerves	-1.54947426	1
Brachial plexus vs. Unspecified plexus	-0.47065279	1
Cranial nerves vs. Unspecified plexus	0.11036353	1
Distal lower extremity nerves vs. Unspecified plexus	-0.87021819	1
Lumbar & sacral nerve roots vs. Unspecified plexus	0.00869611	1
Lumbosacral plexus vs. Unspecified plexus	0	1
Thoracic nerve roots vs. Unspecified plexus	-1.76621913	1
Unspecified peripheral nerves vs. Unspecified plexus	1.54947426	1
Brachial plexus vs. Unspecified spinal nerve roots	-0.95749285	1
Cranial nerves vs. Unspecified spinal nerve roots	-0.56643685	1
Distal lower extremity nerves vs. Unspecified spinal nerve roots	-1.22964552	1
Lumbar & sacral nerve roots vs. Unspecified spinal nerve roots	-0.57783339	1
Lumbosacral plexus vs. Unspecified spinal nerve roots	-0.64654765	1
Thoracic nerve roots vs. Unspecified spinal nerve roots	-1.94530545	1
Unspecified peripheral nerves vs. Unspecified spinal nerve roots	0.4944714	1
Unspecified plexus vs. Unspecified spinal nerve roots	-0.64654765	1

8B

Time from Treatment 1 to Progression

Category & Types of Peripheral Nerves		N	Median (months)	Mean (months)	Standard deviation	Lower bound	Upper bound
Cranial nerves	Cranial nerves	25	9.4	16.042	14.24669318	0	37.89338637
Neural plexus	Brachial plexus	23	36	22.0326087	15.66051497	4.678970052	67.32102995
	Lumbosacral plexus	36	18.9	19.71388889	14.81415159	0	48.52830318
	Unspecified plexus	7	6.4	6.4	9.59342E-16	6.4	6.4
Spinal nerves	Cervical nerve roots	6	3	3.375	2.200852108	0	7.401704215
	Thoracic nerve roots	3	6	5	1.732050808	2.535898385	9.464101615
	Lumbar & sacral nerve roots	10	6	13.783	23.65665236	0	53.31330473
	Unspecified spinal nerve roots	5	11	7.642	4.696894719	1.606210562	20.39378944
Peripheral nerves	Distal upper extremity nerves	1	8	8	N/A	N/A	N/A
	Distal lower extremity nerves	30	11	16.58	12.20518262	0	35.41036524
	Unspecified peripheral nerves	13	9.4	10.41538462	5.468980123	0	20.33796025

N/A = Not applicable

Kruskal-Wallis chi-squared = 21.558	df = 10	p value = 0.01572

Pair	Z score	Bonferroni adjusted p value
Brachial plexus vs. Cervical nerve roots	3.25377157	0.03131805
Cervical nerve roots vs. Lumbosacral plexus	-3.22123907	0.03510035
Cervical nerve roots vs. Distal lower extremity nerves	-3.06746997	0.05936677
Cervical nerve roots vs. Cranial nerves	-2.64497963	0.22466354
Cervical nerve roots vs. Unspecified peripheral nerves	-2.19854113	0.76754059
Brachial plexus vs. Lumbar & sacral nerve roots	2.19076527	0.78289153
Lumbar & sacral nerve roots vs. Lumbosacral plexus	-2.1224255	0.92955563
Brachial plexus vs. Cranial nerves	1.0007963	1
Brachial plexus vs. Distal lower extremity nerves	0.43213429	1
Cranial nerves vs. Distal lower extremity nerves	-0.62551593	1
Brachial plexus vs. Distal upper extremities nerves	0.52857918	1
Cervical nerve roots vs. Distal upper extremities nerves	-0.88104013	1
Cranial nerves vs. Distal upper extremities nerves	0.24592146	1
Distal lower extremity nerves vs. Distal upper extremities nerves	0.41334949	1
Cervical nerve roots vs. Lumbar & sacral nerve roots	-1.28147399	1
Cranial nerves vs. Lumbar & sacral nerve roots	1.44501055	1
Distal lower extremity nerves vs. Lumbar & sacral nerve roots	1.94459022	1
Distal upper extremities nerves vs. Lumbar & sacral nerve roots	0.27639143	1
Brachial plexus vs. Lumbosacral plexus	0.26652966	1
Cranial nerves vs. Lumbosacral plexus	-0.83739636	1
Distal lower extremity nerves vs. Lumbosacral plexus	-0.19667208	1
Distal upper extremities nerves vs. Lumbosacral plexus	-0.46242227	1
Brachial plexus vs. Thoracic nerve roots	2.02442268	1
Cervical nerve roots vs. Thoracic nerve roots	-0.35198134	1
Cranial nerves vs. Thoracic nerve roots	1.56058857	1
Distal lower extremity nerves vs. Thoracic nerve roots	1.85445106	1
Distal upper extremities nerves vs. Thoracic nerve roots	0.6085939	1
Lumbar & sacral nerve roots vs. Thoracic nerve roots	0.62718229	1
Lumbosacral plexus vs. Thoracic nerve roots	1.94956792	1
Brachial plexus vs. Unspecified peripheral nerves	1.17148986	1
Cranial nerves vs. Unspecified peripheral nerves	0.34315339	1
Distal lower extremity nerves vs. Unspecified peripheral nerves	0.86351294	1
Distal upper extremities nerves vs. Unspecified peripheral nerves	-0.12859923	1
Lumbar & sacral nerve roots vs. Unspecified peripheral nerves	-1.00645059	1
Lumbosacral plexus vs. Unspecified peripheral nerves	1.03638094	1
Thoracic nerve roots vs. Unspecified peripheral nerves	-1.30551283	1
Brachial plexus vs. Unspecified plexus	1.78502785	1
Cervical nerve roots vs. Unspecified plexus	-1.29602963	1
Cranial nerves vs. Unspecified plexus	1.12572308	1
Distal lower extremity nerves vs. Unspecified plexus	1.55037329	1
Distal upper extremities nerves vs. Unspecified plexus	0.21569512	1
Lumbar & sacral nerve roots vs. Unspecified plexus	-0.1203193	1
Lumbosacral plexus vs. Unspecified plexus	1.69310771	1
Thoracic nerve roots vs. Unspecified plexus	-0.68421894	1
Unspecified peripheral nerves vs. Unspecified plexus	0.77652708	1
Brachial plexus vs. Unspecified spinal nerve roots	1.46366125	1
Cervical nerve roots vs. Unspecified spinal nerve roots	-1.27055284	1
Cranial nerves vs. Unspecified spinal nerve roots	0.88399188	1
Distal lower extremity nerves vs. Unspecified spinal nerve roots	1.24720308	1
Distal upper extremities nerves vs. Unspecified spinal nerve roots	0.16639277	1
Lumbar & sacral nerve roots vs. Unspecified spinal nerve roots	-0.19646375	1
Lumbosacral plexus vs. Unspecified spinal nerve roots	1.36419144	1
Thoracic nerve roots vs. Unspecified spinal nerve roots	-0.71268229	1
Unspecified peripheral nerves vs. Unspecified spinal nerve roots	0.59997529	1
Unspecified plexus vs. Unspecified spinal nerve roots	-0.08251123	1

8C

Time from Symptom Onset to Diagnosis

**Category & Types of Peripheral Nerves**		**N**	**Median (months)**	**Mean (months)**	**tandard deviatio**	**Lower bound**	**Upper bound**
Cranial nerves	Cranial nerves	26	3	9.014102564	18.82874852	0	40.65749705
Neural plexus	Brachial plexus	40	3	8.08875	13.67470651	0	30.34941302
	Lumbosacral plexus	42	3	9.266666667	20.28016807	0	43.56033613
	Unspecified plexus	0	N/A	N/A	N/A	N/A	N/A
Spinal nerves	Cervical nerve roots	13	5	11.92307692	16.57036279	0	38.14072559
	Thoracic nerve roots	8	5.5	29	42.02380278	0	89.54760556
	Lumbar & sacral nerve roots	17	3	10.70588235	23.05906738	0	49.11813475
	Unspecified spinal nerve roots	0	N/A	N/A	N/A	N/A	N/A
Peripheral nerves	Distal upper extremity nerves	15	3	9.833333333	24.02949576	0	51.05899153
	Distal lower extremity nerves	42	3	9.526984127	17.95227071	0	38.90454143
	Unspecified peripheral nerves	3	6	5.1	3.439476704	0	12.87895341

N/A = Not applicable

Kruskal-Wallis chi-squared = 2.9153 **df = 10** **p value = 0.9396**

**Pair**	**Z score**	**Bonferroni adjusted p value**
Brachial plexus vs. Cervical nerve roots	-1.019157383	1
Brachial plexus vs. Cranial nerves	-0.004628407	1
Cervical nerve roots vs. Cranial nerves	0.954430849	1
Brachial plexus vs. Distal lower extremity nerves	-0.001089024	1
Cervical nerve roots vs. Distal lower extremity nerves	1.024403926	1
Cranial nerves vs. Distal lower extremity nerves	0.003708285	1
Brachial plexus vs. Distal upper extremities nerves	0.171515134	1
Cervical nerve roots vs. Distal upper extremities nerves	0.995688993	1
Cranial nerves vs. Distal upper extremities nerves	0.163754027	1
Distal lower extremity nerves vs. Distal upper extremities nerves	0.173439711	1
Brachial plexus vs. Lumbar & sacral nerve roots	-0.211638901	1
Cervical nerve roots vs. Lumbar & sacral nerve roots	0.716798069	1
Cranial nerves vs. Lumbar & sacral nerve roots	-0.192713302	1
Distal lower extremity nerves vs. Lumbar & sacral nerve roots	-0.212320992	1
Distal upper extremities nerves vs. Lumbar & sacral nerve roots	-0.319560746	1
Brachial plexus vs. Lumbosacral plexus	0.162264543	1
Cervical nerve roots vs. Lumbosacral plexus	1.138113199	1
Cranial nerves vs. Lumbosacral plexus	0.14833142	1
Distal lower extremity nerves vs. Lumbosacral plexus	0.165382881	1
Distal upper extremities nerves vs. Lumbosacral plexus	-0.053458358	1
Lumbar & sacral nerve roots vs. Lumbosacral plexus	0.337867317	1
Brachial plexus vs. Thoracic nerve roots	-1.116484017	1
Cervical nerve roots vs. Thoracic nerve roots	-0.238211558	1
Cranial nerves vs. Thoracic nerve roots	-1.06663965	1
Distal lower extremity nerves vs. Thoracic nerve roots	-1.120317359	1
Distal upper extremities nerves vs. Thoracic nerve roots	-1.10631323	1
Lumbar & sacral nerve roots vs. Thoracic nerve roots	-0.865635327	1
Lumbosacral plexus vs. Thoracic nerve roots	-1.213872044	1
Brachial plexus vs. Unspecified peripheral nerves	-0.348402808	1
Cervical nerve roots vs. Unspecified peripheral nerves	0.18237389	1
Cranial nerves vs. Unspecified peripheral nerves	-0.340125158	1
Distal lower extremity nerves vs. Unspecified peripheral nerves	-0.348580401	1
Distal upper extremities nerves vs. Unspecified peripheral nerves	-0.411864554	1
Lumbar & sacral nerve roots vs. Unspecified peripheral nerves	-0.235191954	1
Lumbosacral plexus vs. Unspecified peripheral nerves	-0.40896969	1
Thoracic nerve roots vs. Unspecified peripheral nerves	0.330656	1

8D

Time from Symptom Onset to Death							
Category & Types of Peripheral Nerves		N	Median (months)	Mean (months)	andard deviatio	Lower bound	Upper bound
Cranial nerves	Cranial nerves	129	10	18.26	28.92546267	0	76.11547314
Neural plexus	Brachial plexus	103	9.996666667	22.69	33.91926048	0	90.52677339
	Lumbosacral plexus	122	12	26.24	40.34105844	0	106.9226907
	Unspecified plexus	27	10	10.88	1.518358688	7.844764105	13.91819886
Spinal nerves	Cervical nerve roots	43	5	14.08	23.64997249	0	61.38320079
	Thoracic nerve roots	30	5.5	18.31	29.34618567	0	77.00303801
	Lumbar & sacral nerve roots	62	7.78	18.25	22.92419097	0	64.09951097
	Unspecified spinal nerve roots	33	10	17.17	31.4554133	0	80.08234175
Peripheral nerves	Distal upper extremity nerves	31	5	13.28	24.09619209	0	61.46980353
	Distal lower extremity nerves	125	8	22.58	35.86550833	0	94.30984333
	Unspecified peripheral nerves	80	10	11.09	11.43378045	0	33.95793589

Kruskal-Wallis chi-squared = 33.352	df = 10	p value = 0.1534
Pair	Z score	Bonferroni adjusted p value
Distal upper extremities nerves vs. Unspecified plexus	-2.56858453	0.2808157
Cervical nerve roots vs. Unspecified plexus	-2.51412714	0.3281504
Brachial plexus vs. Distal upper extremities nerves	2.29690682	0.5946624
Distal upper extremities nerves vs. Lumbosacral plexus	-2.29141518	0.6033338
Brachial plexus vs. Cervical nerve roots	2.26766473	0.6421154
Cervical nerve roots vs. Lumbosacral plexus	-2.26706929	0.6431148
Brachial plexus vs. Cranial nerves	0.60944676	1
Cervical nerve roots vs. Cranial nerves	-1.88079112	1
Brachial plexus vs. Distal lower extremity nerves	0.78640823	1
Cervical nerve roots vs. Distal lower extremity nerves	-1.73688761	1
Cranial nerves vs. Distal lower extremity nerves	0.19217326	1
Cervical nerve roots vs. Distal upper extremities nerves	0.24964284	1
Cranial nerves vs. Distal upper extremities nerves	1.94979701	1
Distal lower extremity nerves vs. Distal upper extremities nerves	1.82357256	1
Brachial plexus vs. Lumbar & sacral nerve roots	0.30247953	1
Cervical nerve roots vs. Lumbar & sacral nerve roots	-1.82962067	1
Cranial nerves vs. Lumbar & sacral nerve roots	-0.20649498	1
Distal lower extremity nerves vs. Lumbar & sacral nerve roots	-0.36070167	1
Distal upper extremities nerves vs. Lumbar & sacral nerve roots	-1.91806839	1
Brachial plexus vs. Lumbosacral plexus	0.0721824	1
Cranial nerves vs. Lumbosacral plexus	-0.56119959	1
Distal lower extremity nerves vs. Lumbosacral plexus	-0.74640169	1
Lumbar & sacral nerve roots vs. Lumbosacral plexus	-0.24980988	1
Brachial plexus vs. Thoracic nerve roots	1.28906142	1
Cervical nerve roots vs. Thoracic nerve roots	-0.60653007	1
Cranial nerves vs. Thoracic nerve roots	0.92209766	1
Distal lower extremity nerves vs. Thoracic nerve roots	0.80069236	1
Distal upper extremities nerves vs. Thoracic nerve roots	-0.79303814	1
Lumbar & sacral nerve roots vs. Thoracic nerve roots	0.98387546	1
Lumbosacral plexus vs. Thoracic nerve roots	1.26492143	1
Brachial plexus vs. Unspecified peripheral nerves	0.55936367	1
Cervical nerve roots vs. Unspecified peripheral nerves	-1.73651238	1
Cranial nerves vs. Unspecified peripheral nerves	0.01987302	1
Distal lower extremity nerves vs. Unspecified peripheral nerves	-0.14870224	1
Distal upper extremities nerves vs. Unspecified peripheral nerve	-1.8301105	1
Lumbar & sacral nerve roots vs. Unspecified peripheral nerves	0.20531038	1
Lumbosacral plexus vs. Unspecified peripheral nerves	0.51229717	1
Thoracic nerve roots vs. Unspecified peripheral nerves	-0.85982011	1
Brachial plexus vs. Unspecified plexus	-0.95099977	1
Cranial nerves vs. Unspecified plexus	-1.3520748	1
Distal lower extremity nerves vs. Unspecified plexus	-1.46199754	1
Lumbar & sacral nerve roots vs. Unspecified plexus	-1.10259776	1
Lumbosacral plexus vs. Unspecified plexus	-1.01217783	1
Thoracic nerve roots vs. Unspecified plexus	-1.78324837	1
Unspecified peripheral nerves vs. Unspecified plexus	-1.29835228	1
Brachial plexus vs. Unspecified spinal nerve roots	-0.12989041	1
Cervical nerve roots vs. Unspecified spinal nerve roots	-1.89131243	1
Cranial nerves vs. Unspecified spinal nerve roots	-0.54600795	1
Distal lower extremity nerves vs. Unspecified spinal nerve roots	-0.66747467	1
Distal upper extremities nerves vs. Unspecified spinal nerve root	-1.98511661	1
Lumbar & sacral nerve roots vs. Unspecified spinal nerve roots	-0.34621549	1
Lumbosacral plexus vs. Unspecified spinal nerve roots	-0.18164253	1
Thoracic nerve roots vs. Unspecified spinal nerve roots	-1.16314599	1
Unspecified peripheral nerves vs. Unspecified spinal nerve roots	-0.52850393	1
Unspecified plexus vs. Unspecified spinal nerve roots	0.69222283	1
